# A leucine responsive small RNA AbcR200 regulates expression of the lactate utilization (lut) operon in *Acinetobacter baumannii* DS002

**DOI:** 10.1016/j.jbc.2025.108160

**Published:** 2025-01-10

**Authors:** Harshita Nagasai Yakkala, Ashok Kumar Madikonda, Sandhya Rani Behera, Vijaykumar Pillalamarri, Kashif Gulam Mohammad, Ganeshwari Dhurve, Prasad Tammineni, Suresh Babu Pakala, Dayananda Siddavattam

**Affiliations:** 1 Department of Animal Biology, School of Life Sciences, University of Hyderabad, Hyderabad, India; 2 Department of Biochemistry, School of Life Sciences, University of Hyderabad, Hyderabad, India

**Keywords:** bacterial small RNAs, lactate utilization, leucine responsive regulatory protein, gene expression, post transcriptional regulation

## Abstract

Noncoding small RNAs are essential for modulating bacterial gene expression, especially under carbon and nutrient-limited conditions. In this study, by using both *in silico* and molecular hybridization tools, we identified a carbon source responsive small RNA in *Acinetobacter baumannii* DS002. Expression of corresponding gene, *abcR200*, located at the intergenic region of *omt* (O-methyl transferase) and *orf72* genes, is under the transcriptional control of a global transcriptional factor, leucine responsive regulatory protein (Lrp). A sequence motif that serves as a target for Lrp was found overlapping the *abcR200* promoter (*P*_*abcR200*_). Chromatin immunoprecipitation demonstrated that Lrp oligomers, formed under low leucine conditions, strongly interacted to the *P*_*abcR200*_. However, the observed interactions were disrupted in the presence of leucine, as leucine promoted dissociation of Lrp to monomers and dimers, the conformation unfavorable to interact with *P*_*abcR200*._ The *abcR200* promoter activity increased with increase of exogenous leucine concentrations, and at 2 mM leucine concentration, maximum promoter activity was observed. The *AbcR200* target mRNAs were identified by analyzing the transcriptome of *abcR200* negative strain of *A. baumannii*. Intriguingly, in *abcR200* negative background, expression of *lut* (lactate utilization) mRNA has increased, suggesting *lut* mRNA as one of the mRNA targets for *AbcR200*. Consistent of this observation, there existed extensive sequence complementarity between *AbcR200* and *lut* mRNA, especially in the regions coding LutP, LutE, and LutR. In support of the observed sequence complementarity, the levels of *lut* mRNA encoded proteins got elevated in *abcR200* negative HS002 strains suggesting a role for *AbcR200* in translational inhibition of *lut* mRNA.

Genus *Acinetobacter* includes free-living saprophytic bacterial species found in soil, water, sewage, and foods. These ubiquitous organisms are also isolated as commensals from the skin of animals and humans, especially in staff working in hospitals and patients (1, 2, 3). Among them, *Acinetobacter baumannii* strains are opportunistic pathogens. They account for a significant portion of hospital-acquired infections caused by gram-negative bacteria (4). Before 1970s, *A. baumannii* strains were sensitive to most of the antimicrobials. Antibiotics like gentamicin, ampicillin, and nalidixic acid have effectively controlled *A. baumannii* infections (5, 6, 7). However, by late 1970s, they started showing resistance to most of the commonly used antibiotics. Soon, a pan drug-resistant strain of *A. baumannii* was isolated from hospital settings (8, 9, 10). *A. baumannii* strains exhibit a greater degree of genome plasticity (11, 12, 13). They acquire genetic material through conventional horizontal gene transfer or through membrane vesicles (14, 15, 16, 17, 18, 19). Intergenomic mobility of resistance genes is quite evident in certain strains of *A. baumannii*. The 89 kb resistance island identified in the genome of AYE strain contains forty-five drug-resistant genes. Most of them are orthologous to the resistance genes identified in *Pseudomonas*, *Salmonella*, and *Escherichia coli* (20).

The strains of *A. baumannii* are highly adaptable to a variety of ecological niches. They survive in soil, water, and on the surface of hospital ware as free-living organisms and quickly adapt to the pathogenic lifestyle (21, 22, 23). The ability to thrive on various carbon sources is assumed to be the reason behind the robust lifestyle of the strains (21). Most of the *A. baumannii* strains prefer succinate/acetate as a source of carbon. However, quite a few survive on disinfectants, recalcitrant xenobiotics, and phenolic compounds (24, 25, 26, 27).

Our laboratory isolated the *A. baumannii* strain DS002 from pesticide contaminated agricultural soils and sequenced its genome to gain better understanding on its biodegradation potential (23, 28). Similar to environmental strains of *A. baumannii* that exhibit a diauxic growth pattern (29), the DS002 genome contains genes encoding novel monooxygenases and dioxygenases, essential for initiating the degradation of several aromatic compounds (23). Notably, these genes are exclusively present in environmental isolates and are absent in clinical isolates (23). This selective enrichment of degradative genes, combined with distinct regulatory mechanisms, likely enhances the adaptive potential of environmental isolates of *A. baumannii*.

Bacterial small RNAs regulate a variety of biological processes through binding to their target mRNAs and regulatory proteins (30, 31, 32, 33, 34, 35). The regulatory roles of small RNAs are well studied in model organisms such as *E. coli*, *Salmonella enterica*, and, *Staphylococcus aureus* (36, 37, 38, 39, 40, 41, 42, 43, 44, 45, 46, 47, 48, 49). However, despite its clinical and environmental relevance, similar studies are scarce in *A. baumannii* (50, 51). In this study, we analyzed the genome sequence of *A. baumannii* strain DS002, which has demonstrated the ability to use recalcitrant aromatic compounds as sole carbon source (23), to identify small RNAs involved in regulation of carbon metabolism. Our investigations uncovered a small RNA whose expression is regulated by the transcription factor, leucine responsive regulatory protein (Lrp). One of the key targets of this Lrp-regulated small regulatory RNA (sRNA) is the mRNA of the lactate utilization (*lut*) operon. Findings of this study highlight the intricate regulatory role of leucine responsive sRNA in regulating lactate metabolism in *A. baumannii* DS002.

## Results

### Carbon responsive small RNAs

**Figure 1:**
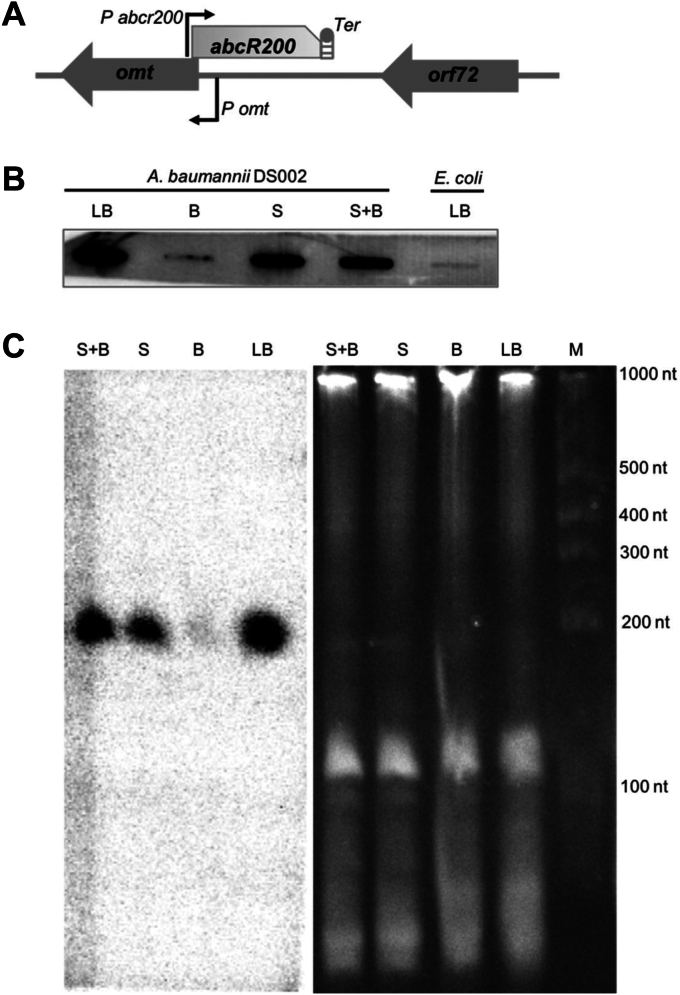
**Expression of *abcR200* in response to different carbon sources.***A*, Organization of *abcR200* gene in *A. baumannii* DS002. *B*, Slot blot analysis showing the expression of *abcR200* in LB (LB), benzoate (B), succinate (S) and succinate and benzoate (S + B). The total RNA isolated from *E. coli* (lane E) served as negative control. *C*, Northern blot analysis showing the expression and size of *AbcR200* in *A. baumannii* DS002 cells grown in succinate and benzoate (S + B), succinate (S), benzoate (B), and LB (LB).

Primary objective of this study was to identify small RNAs of *A. baumannii* DS002 involved in regulation of carbon catabolism. Initially, we have used *in silico* tools to identify small RNA coding sequences in *A. baumannii* DS002 genome (52). sRNA Identification protocol using high-throughput technologies (SIPHT) predicted existence of only 14 small RNA coding genes in the genome of *A. baumannii* DS002, whereas sRNA scanner predicted existence of as many as 266 small RNA coding genes. The predicted sRNA coding gene sequences (266 + 14) were then used as input to identify sequence motifs that serve as targets to the transcription factors involved in carbon catabolism. Interestingly, we predicted presence of consensus Lrp binding site overlapping the promoter region of four sRNA coding genes. Initially, we performed slot blot analysis to gain quick assessment on the expression pattern of these four small RNA coding genes (Figs. 1, *A* and *B* and S1). Total RNA isolated from the cells grown in nutrient rich (LB), preferred (succinate) and less preferred (benzoate) carbon sources were probed by the end labeled oligos complementary to the predicted sRNAs. Out of these four small RNA coding genes, only the one identified in the intergenic region of O-methyl transferase (omt) and *orf72* genes (Fig. 1*A*) has shown clear carbon specific expression pattern (Fig. 1*B*). It was significantly upregulated in cultures grown in nutrient rich LB medium (Fig. 1*B*, lane LB) and completely repressed in cultures grown in minimal medium containing less preferred carbon source, benzoate (Fig. 1*B*, lane B). However, its expression was restored in cultures grown either in succinate or in cultures gown in the medium containing both succinate and benzoate (Fig. 1*B*, lanes S and S + B). This carbon responsive small RNA was given a four-letter code name, AbcR, (*A. baumannii*, “c” for carbon, “R” for responsive) followed by a number that indicates its predicted size. Since the predicted size of the small RNA is approximately 200 bases, it is designated as *AbcR200*. After gaining preliminary assessment on the expression pattern of *abcR200*, we have performed Northern blots to revalidate the predicted size and expression profile of *abcR200* (Fig. 1*C*). Reconfirming the results obtained from slot blot analysis, the Northern blots have shown very low levels of *abcR200* expression in cultures grown in the presence of benzoate (Fig. 1*C*, lane B). However, a prominent *AbcR200* specific signal was detected in cultures grown in succinate (Fig. 1*C*, lane S) and succinate and benzoate (Fig. 1*C*, lane S + B), and its intensity has further increased in cultures grown in LB medium (Fig. 1*C*, lane LB). The size of the *AbcR200* specific signal matched with the mobility of the marker RNA with a size of 200 nucleotides and it coincided with the predicted size of *AbcR200*.

### Regulation of *abcR200*

**Figure 2:**
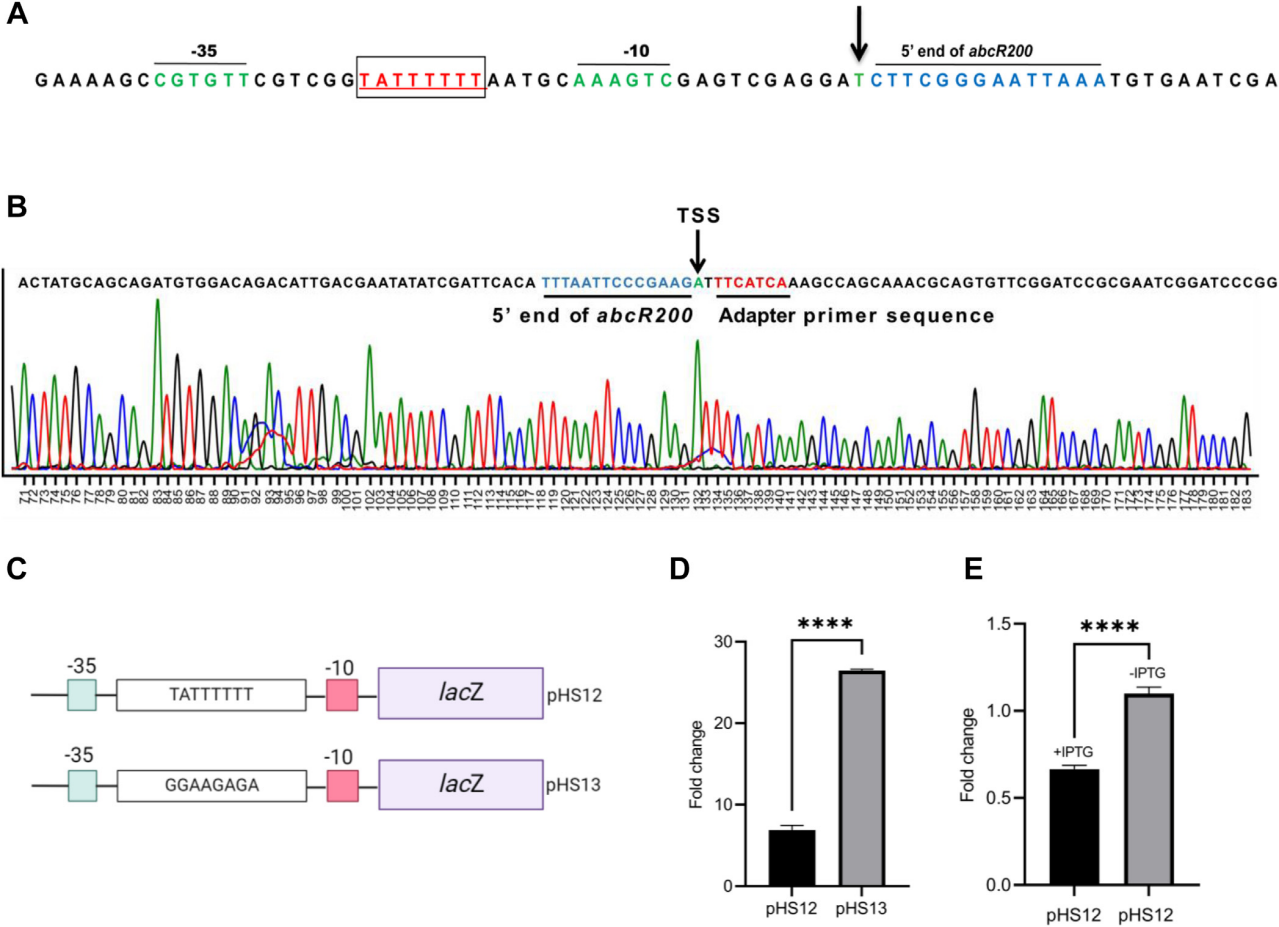
**Lrp regulates the expression of *abcR200* gene.***A*, Sequence of the 5′ region of *abcR200* gene. Transcription start site (TSS) is shown with *inverted arrow*. The −10 and −35 promoter hexamers are shown with *green color font*. Predicted Lrp-binding site shown in *red color font* is *underlined* and indicated in an *open box*. *B*, The sequence of the 5′ RACE product. The TSS is shown with an *inverted arrow*, the adapter (*green color* font) and the sequence of 5′ region of *AbcR200* (*red color* font) is *underlined*. *C*, Model depicting the construction of *abcR200* promoter *lacZ* fusions with WT (pHS12) and mutant (pHS13) Lrp binding sites. *D*, β-galactosidase activity levels in *lrp* positive AM001 cells carrying *abcR200* promoter *lacZ* fusions, with WT (pHS12) and mutant (pHS13) Lrp binding sites. *E*, β-galactosidase activity levels in *Escherichia coli* HS001 (pHS11 + pHS12) grown in the presence and absence of IPTG. Data plotted are mean ± SD of three independent experiments. Statistical significance of data wherever applicable is indicated by ∗*p* < 0.05; ∗∗∗*p* < 0.001. Lrp, leucine responsive regulatory protein; RACE, rapid amplification of cDNA ends.

As stated before, the *abcR200* gene is identified in the intergenic region of *omt* and *orf72* genes (Fig. 1*A*). The *omt* gene found upstream of *abcR200* codes for O-methyl transferase, whereas the gene (*orf72*) identified downstream of *abcR200* codes for a protein of an unknown function (Fig. 1*A*). In fact, the *abcR200* has opposite transcription orientation when compared to the transcription of *omt* gene (Fig. 1*A*). The putative promoter motif of the *abcR200* (P_*abcR200*_) is identified at the 5′ end of the *omt* gene and hence the *abcR200* overlaps with the 5′ region of *omt* gene (Fig. 1*A*). The functional status of predicted P_*abcR200*_ was validated by performing both *in vitro* and *in vivo* studies. Initially, we have performed 5′ rapid amplification of complementary DNA (cDNA) ends (RACE) to determine transcription start site (TSS) of *abcR200*. The sequence of the 150 bp RACE product contained adaptor sequence followed by a sequence identical to the 5′ region of *abcR200* gene (Fig. 2*B*). The sequence of *abcR200* started with an adenine residue, indicating it as the TSS of *abcR200* gene (Fig. 2, *A* and *B*). The promoter hexamers identified at −10 (CGGTAT) and −35 (TTGAAA) regions have shown similarities with the consensus σ^70^ dependent promoters (53). Furthermore, a sequence motif that showed significant identity with the consensus Lrp binding site (TATTTTTT) was identified overlapping the promoter motif of *abcR200* gene (Fig. 2*A*). We have performed both genetic and biochemical assays to validate the functional status of Lrp binding site. Initially, we have constructed *abcR200*-*lacZ* fusions by including the WT (pHS12), and mutant (pHS13) Lrp binding sites (Fig. 2*C*) and then electroporated them into *lrp* positive *E. coli* AM001 cells. While growing *lrp* positive *E. coli* AM001 (pHS13) cells in nutrient rich LB medium, we observed a fourfold increase in β-galactosidase activity (Fig. 2*D*) compared to *E. coli* AM001 (pHS12) cells containing the *abcR200-lacZ* fusion with a WT *lrp* binding site (Fig. 2*D*). The repressive *lrp* positive genetic background and growth condition did not influence the promoter activity when mutations were introduced in the consensus Lrp binding site of *abcR200* gene (Fig. 2*D*). These results were further validated by creating *lrp* negative genetic background in *E*. *coli*. The *E. coli lrp* negative (HS001) cells were then cotransformed with an expression plasmid, pHS11 coding *A. baumannii* DS002 Lrp protein (_*Ab*_Lrp^C6His^) from an IPTG inducible promoter along with an *abcR200-lacZ* fusion (pHS12) having WT Lrp binding site. The *E. coli* HS001 (pHS11+ pHS12) cells were then grown in nutrient rich LB medium both in the presence and absence of IPTG. Reconfirming the repressive role of Lrp, the *abcR200* promoter was active only when the expression of _*Ab*_Lrp^C6His^ was not induced (Fig. 2*E*, lane (-IPTG)). However, there was a significant reduction in the activity of *abcR200* promoter (Fig. 2*E*, lane (+IPTG)) when expression of _*Ab*_Lrp^C6His^ was induced by adding IPTG.

### Lrp oligomers bind to P_*abcR200*_

**Figure 3:**
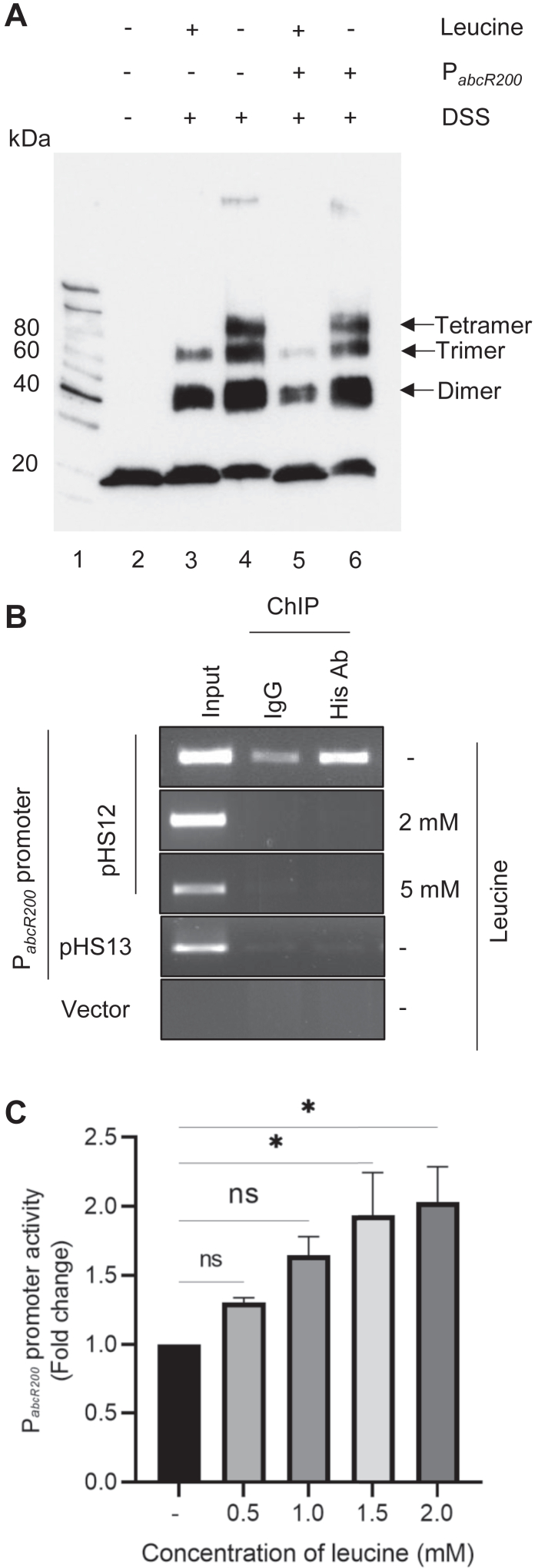
**P*abcR200* and**_***Ab***_**Lrp**^**C6His**^**interactions.***A*, Leucine induced conformational changes in _*Ab*_Lrp^C6His^. Pure _*Ab*_Lrp^C6His^ (lane 2) incubated in the presence (lane 3) and absence (lane 4) of leucine was cross-linked using DSS (disuccinimidyl suberate) and separated on 12.5% SDS-PAGE, and the Western blot analysis was performed by probing with anti-His antibodies. The _*Ab*_Lrp^C6His^ dimers, trimers, and tetramers formed are shown with *arrows*. Formation of _*Ab*_Lrp^C6His^ tetramers is seen both in the absence (lane 4) and presence of P*abcR200* (lane 6). Lane 1 represents the protein marker. *B*, The ChIP assays: The *Escherichia coli* HS001 (pHS11 + pHS12) grown in the absence and presence of leucine (2 mM and 5 mM) were induced to express _*AB*_LRP^C6His^. The clear lysate prepared from these cells were immunoprecipitated (IP) using anti-His antibodies, and the obtained precipitates were used to amplify P*abcR200*. P*abcR200* amplification was only seen in cells grown in the absence of leucine, and no such amplification was observed in cells grown in the presence of leucine. Cell lysates prepared *E. coli* HS001 (pHS11 + pHS13), *E. coli* HS001 (pHS11 + pMP220) served as negative controls. *C*, Promoter assays were performed by measuring β-galactosidase activity levels in cells of *E. coli* HS001 (pHS11 + pHS12) cells grown in the absence and presence of 0. 5, 1.0, 1.5, and 2.0 mM leucine. Promoter activity increased with the increase of leucine concentration, and maximum activity was observed in cells grown in media containing 2.0 mM leucine. One-way Anova was used to plot the data and it represents mean ± SD of three independent experiments. Statistical significance of data wherever applicable is indicated by ∗*p* < 0.05; ∗∗∗*p* < 0.001. Lrp, leucine responsive regulatory protein; ChIP, chromatin immunoprecipitation.

Lrp exerts its influence over a number of bacterial genes by responding to intracellular leucine concentrations (54, 55, 56). When leucine concentration is low, Lrp adopts a high affinity DNA-binding oligomeric state. Conversely, in the presence of leucine, it dissociates into a transcriptionally inactive monomeric or dimeric state (54, 56). Since promoter assays indicated a potential role for _*Ab*_Lrp^C6His^ in regulating *abcR200* gene expression (Fig. 2*E*), further studies were conducted to determine whether intracellular leucine concentration influences the regulation of *abcR200*. This proposed regulatory role of leucine was validated by performing both *in vitro* and *in vivo* experiments. We initially performed *in vitro* studies to investigate whether _*Ab*_LRP^C6His^ oligomers form in the absence of leucine, and to assess if their formation is dependent on the *abcR200* (*P*_*abcR200*_) promoter. The pure _*Ab*_Lrp^C6His^ (Fig. S2) incubated with a cross linker disuccinimidyl suberate (DSS), was resolved on SDS-PAGE (12.5%) and formation of _*Ab*_Lrp^C6His^ oligomers were detected by performing Western blots using anti-His antibodies (Fig. 3*A*). As expected, leucine promoted dissociation of _*Ab*_Lrp^C6His^ into dimeric and trimeric states (Fig. 3*A* lanes 3 & 5). The tetrameric _*Ab*_Lrp^C6His^ was only seen in reactions performed in the absence of leucine (Fig. 3*A*, lanes 4 & 6). Intriguingly, the DNA fragment containing the *abcR200* promoter with the Lrp binding site had no effect on the formation of _*Ab*_Lrp^C6His^ tetramers, suggesting that the absence of leucine alone drives the formation of these tetramers (Fig. 3*A* lanes 4 & 6). After confirming the formation of _*Ab*_Lrp^C6His^ oligomers in the absence of leucine, we conducted chromatin immunoprecipitation (ChIP) analysis to determine whether these Lrp oligomers interact with the *abcR200* promoter. The cell lysate prepared from a *lrp* negative *E. coli* HS001 strain containing an *abcR200-lacZ* fusion (pHS12) and a compatible expression plasmid (pHS11) coding _*Ab*_Lrp^C6His^ was immunoprecipitated using anti-His antibodies and the precipitate was then used to detect P_*abcR200*_. The ChIP assays convincingly demonstrated recruitment of _*Ab*_Lrp^C6His^ onto P_*abcR200*_ only in the *E. coli* cells grown in the absence of leucine (Figs. 3*B* and S3). Immunoprecipitates prepared from lysates of *E. coli* HS001 (pHS11 + pHS12) cells grown in the presence of exogenous leucine (2 mM and 5 mM) no such promoter recruitment was observed (Figs. 3*B* and S3). Revealing the specificity of _*Ab*_Lrp^C6His^ and P_*abcR200*_ interactions, no P_*abcR200*_ promoter was detected in immunoprecipitates performed using lysates of *E. coli* cells (pMMB206 +pHS11) containing expression vector and *abcR200*-*lacZ* fusion (Fig. 3*B*), or in cells having expression plasmid (pHS11) coding _*Ab*_Lrp^C6His^ and *abcR*-*lacZ* fusion (pHS13) with mutant *lrp* binding site (Figs. 3*B* and S3). The results of the ChIP assays, when considered alongside *in vitro* studies suggesting the formation of Lrp oligomers in the absence of leucine (Fig. 3*A*), strongly indicate that Lrp, in its oligomeric state, interacts with the Lrp-binding motif located within the promoter region of the *abcR200* gene (Fig. 3*B*). Finally, we have performed promoter assays in *E. coli* HS001 (pHS12) cells to assess the influence of exogenous leucine on the expression of *abcR200* gene. The *E. coli* cells (pHS12) were grown in the presence of different concentrations of exogenous leucine, and promoter assays were performed by measuring β-galactosidase activity (Fig. 3*C*). The promoter activity, as determined by measuring β-galactosidase activity, increased with an increase in exogenous leucine concentration and showed maximum activity at 2 mM (Fig. 3*C*). These results, together with the results of ChIP assays, clearly demonstrate the role of Lrp in regulating the expression of *abcR200* gene by modulating its oligomeric state in response to exogenous leucine concentration.

### Lactate utilization (*lut*) operon is part of *abcR200* regulon

Bacterial sRNAs modulate expression of a multitude of genes by exerting their influence through the sequestration of regulatory proteins or direct interaction with target mRNAs (49). In this study, we report existence of a Lrp regulated small RNA coding gene, *abcR200*, in *A. baumannii* DS002. Lrp, is widely regarded as feast/famine regulatory protein and directly controls expression of significant number of genes that perform wide variety of functions in Gram negative bacteria (56). Intriguingly, in *A*. *baumannii*, Lrp is regulating the expression of a regulatory small RNA, *AbcR200*, and therefore expected to exert indirect influence on *A. baumannii* gene expression. We have performed additional experiments to unravel this regulatory interplay by identifying the mRNA targets of *AbcR200*. Initially we have generated *abcR200* negative genetic background in *A. baumannii* DS002 and the *abcR200* negative strain, HS002 was then used to determine the transcription profile by RNA sequence analysis (RNA sequence accession number, SRA: SAMN32537938; ID: 26171994). The generated transcription profile was then compared with the transcriptome of WT strain DS002 grown under identical growth condition. Approximately, 12 to 24 million raw reads were generated independently for the total RNA isolated from three biological replicates of WT and *abcR200* negative *A. baumannii* HS002 strain (Table S1). These raw reads were then used to identify the list of differentially expressed genes by setting the cutoff value of p.adj <0.5 and log2 fold change greater than 1. Analysis of the sequence data following the set rigorous parameters led to the identification of 123 differentially expressed genes within the *abcR200* null background, of which 66 and 57 genes were downregulated and upregulated, respectively (Tables S2 and S3). As shown in logarithmic plot, genes involved in *lut* were found in the list of significantly upregulated genes (Fig. S4 and Table S3). Lactate permease (*lut*P), transcriptional regulator (*lut*R), D-lactate dehydrogenase (*lut*E), and α-hydroxy-acid oxidizing enzyme (*lut*D) are all upregulated in *abcR200* negative background, suggesting a repressive role of *abcR200* on expression of *lut* genes.

### The *lut* mRNA is complementary to *AbcR200*

As described in methods section, deletion of *abcR200* invariably disrupts the *omt* gene. Consistent of the genetic deletion, the *omt* gene was also found in the list of downregulated genes in *A. baumannii* HS002 cells (Fig. S4 and Table S2). Therefore, the observed expression profile, especially the upregulation of *lut* gene expression was validated by performing additional experiments. As small RNAs influence gene expression by directly interacting with target mRNAs, we initially performed *in silico* studies to predict sequence complementarity between *lut* mRNA and *AbcR200*. These *in silico* predictions were then validated by quantifying *lut* mRNA in *omt* positive and *abcR200* negative genetic background.

**Figure 4:**
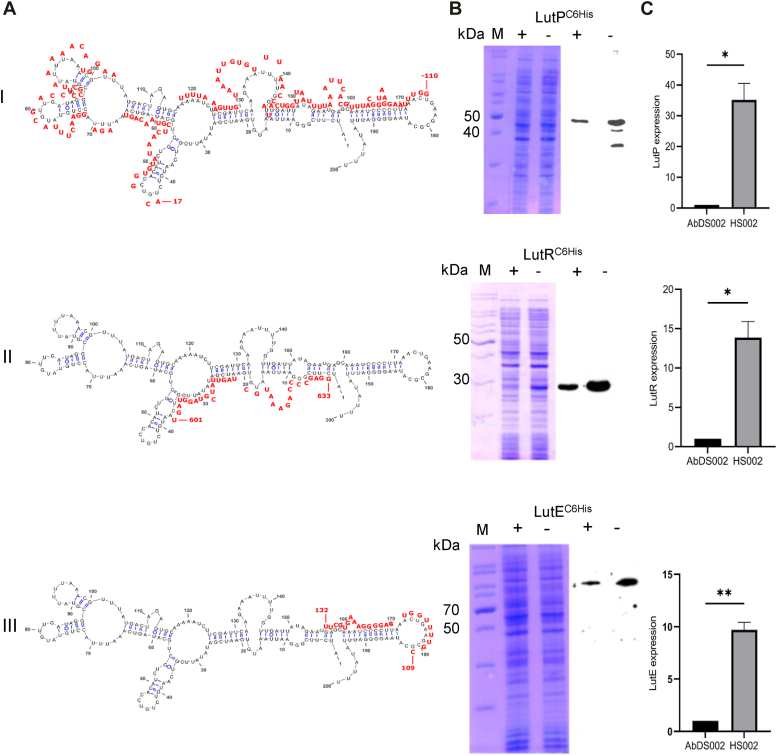
**Base pairing regions between and*****lut mRNA*****.***A*, Indicates predicted complementary regions between *AbcR200* (*black font*) and *lut* mRNA (*red font*) corresponding to *lutP* (panel A, row-I), *lutR* (panel A, row-II), and *lutE* (panel A, row-III). Both negative and positive numbering is given to the *lut* mRNA sequences complementary to *AbcR200*. The “A” of start codon ATG is numbered with +1. The sequence upstream of it is shown with negative numbering, whereas downstream sequence is given positive numbering. *B*, SDS-PAGE and corresponding Western blots indicate ectopically expressed Lut proteins from an IPTG inducible promoter. The WT *A. baumannii* DS002 and *abcR200* negative HS002 cells were transformed with expression plasmids pHS8, pHS7, and pHS6 coding LutP^C6His^, LutR^C6His^, and LutE^C6His^, respectively and their expression was monitored by performing Western blot analysis using anti-His antibodies. Lane 1 indicates molecular size markers, lanes + and – represent total proteins isolated from the WT DS002 and *abcR200* negative HS002 cells of *A. baumannii* carrying plasmid pHS8 (row-I), pHS7 (row-II), and pHS6 (row-III). *C*, The *omt* positive and *abcR200* negative genetic background was generated by transforming plasmid pHS14, coding O-methyl transferase (Omt) from an IPTG inducible promoter into *A. baumannii* HS002 cells. The total RNA isolated from *A. baumannii* HS002 (pHS14) and WT *A. baumannii* DS002 cells was used for the quantification of *lut* mRNA by performing qPCR. Graph represents concentration of *LutP* (row-I), *LutR* (row-II), and *LutE* (row-III) specific mRNAs in *abcR200* positive (lane I) and negative (lane II) backgrounds. LutP, lactate permease; qPCR, quantitative PCR.

Generally, extensive sequence complementarity exists between *cis*-coded small RNAs and their target mRNAs (31). The *trans*-encoded small RNAs exert regulatory effect by establishing short imperfect base pairing with the target gene mRNAs (31). The *AbcR200* is a *trans*-coded small RNA to *lut* operon. There exists 1.1 Mb distance between *lut* operon and *abcR200* gene (23). Surprisingly, extensive sequence complementarity was noticed between various regions of *lut* mRNA and *AbcR200* (Fig. 4*A*, rows I-III). Notably, a 134 bases long region having potential to form imperfect base pairing was found between *AbcR200* and the 5′UTR of *lutP*. The sequence complementarity started from −119 region and it continued till +15, where “A” of start codon ATG is taken as +1 (Fig. 4*A*, row I). Due to existence of intra strand complementarity, especially between 5′ and 3′ regions, the *AbcR200* sequence acquires an overall hair-pin loop structure with several stems and loops (Fig. 4*A*). The sequence of *AbcR200* (43–182) that shows complementarity to *lutP* mRNA exists at the arm-II of the stem structure (Fig. 4*A*, row I). Similar interacting regions were found between *AbcR200* and *lut* mRNA regions coding LutE, and LutR. However, they were found either near TSS of *lutE* or at the 3′ end of the coding region of LutR (Fig. 4*A*, rows II & III). The sequence found at the arm I (3–60) of *AbcR200* showed complementarity with these regions. The length of interacting regions and predicted binding energies was determined following online tool IntaRNA 2.0, and was sufficiently strong to exert translational inhibition on *lut* mRNA (57).

### *AbcR200* inhibits translation of *lut* mRNA

These *in silico* predictions were further validated by monitoring the expression of *lut* genes both in WT (DS002) and in HS002 cells of *A. baumannii*. Initially, we have constructed broad host range mobilizable expression plasmids coding LutR^C6His^ (pHS6), LutE^C6His^ (pHS7), and LutP^C6His^ (pHS8) by including the predicted interacting regions. Construction of such expression plasmids was possible as sufficient length of predicted interacting regions with *AbcR200* was found down stream of translational initiation codon ATG (Fig. 4*A*, rows I, II, and III). These expression plasmids were then independently mobilized into WT (DS002) and *abcR200* negative strain (HS002) of *A. baumannii*. The expression levels of these ectopically expressed proteins were monitored by probing with anti-His antibody. As expected, signals that matched with the predicted size of LutP, LutE, and LutR were detected in both WT and *abcR200* negative cells (Fig. 4*B*, rows I, II, and III). However, the expression levels of LutP, LutE, and LutR were markedly lower in WT cells compared to their expression levels in HS002 cells, implying a negative impact of *AbcR200* on the expression of these genes (Fig. 4*B*, rows I, II, and III, lanes indicated with negative ‘−’ sign). The faint signals observed in WT cells may be attributed to the induction of ectopically expressed *lut* genes, which were cloned under the control of a strong inducible promoter (Fig. 4B, rows I, II, and III, lanes indicated with positive “+” sign). Conversely, in HS002 cells, the absence of *AbcR200* mediated translational inhibition resulted in significantly elevated expression levels of LutP, LutE, and LutR (Fig. 4*B*, rows I, II, and III). Multiple signals were noticed in HS002 cells expressing LutP, probably due to degradation of membrane associated LutP in cells grown in physiologically unfavorable condition for LutP expression (Fig. 4*B*, row I). The elevated expressions of proteins coded by *lut* genes in HS002 cells clearly support *in silico* predictions which suggest possible *AbcR200* mediated translational inhibition of *lut* mRNA.

**Figure 5:**
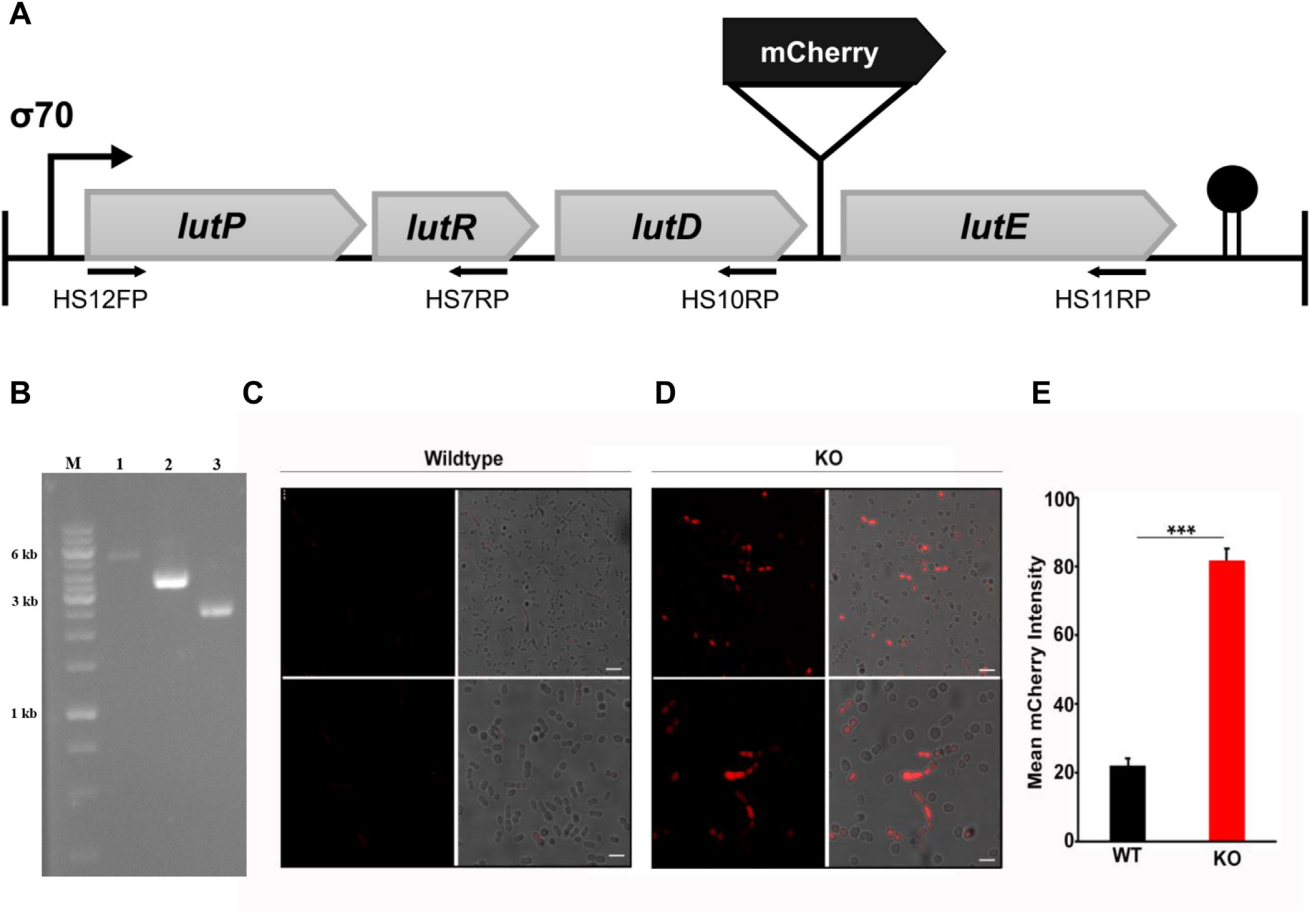
**Confocal Studies.***A*, Organization of *lut* operon in *A. baumannii* DS002. Putative promoter and terminator motifs of *lut* operon are shown with *bent arrow* and *solid circle*, respectively. *Black solid arrow* indicates ORF coding mChery inserted at the intergenic region of *lutD* and *lutE*. *Solid lines* below ORFs indicate sequence regions used while designing primers for performing RT-PCR. *B*, Agarose gel (0.8%) showing the size of amplicons generated in RT-PCR reactions. Lane M indicates molecular size markers, lanes 1 to 3 show amplicons obtained in an RT-PCR mix containing HS12FP as fixed forward and HS11RP (lane 1), HS10RP (lane 2), and HS7RP (lane 3) as reverse primers. *C* and *D*, Confocal images were obtained for *abcR200* positive DS002 (pPS5) and negative HS002 (pPS5) cells of *A. baumannii*, respectively. Representative images were picked from three biological replicates. Each experiment had roughly 1000 cells for quantification. To be precise, the number of WT cells are 3760 and KO cells are 3047. *E*, Graph representing the mean fluorescence intensity obtained for *abcR200* positive DS002 (pPS5) and negative HS002 (pPS5) *A. baumannii* cells. Intensity values plotted come from the average, and error bars represent the standard deviation. Student *t* test was done for *p*-values.

The aforementioned study refers to the expression of *lut* specific ORFs from an inducible promoter. In *A. baumannii* DS002 cells, the 5.4 kb *lut* cluster encompassing *lutP, lutR, lutD*, and *lutE* genes showed an operon-like organization (23). A consensus σ^70^ promoter is predicted upstream of the *lutP* gene and a putative Rho-dependent transcription terminator like motif is only seen downstream of the *lutE* gene (Fig. 5*A*). Absence of promoter and terminator motifs flanking *lutR* and *lutD* ORFs favors organization of *lut* gene cluster as one transcriptional unit. These *in silico* predictions were further validated by measuring the size of *lut* mRNA by performing RT-PCR. The *lut* mRNA was reverse transcribed by using a fixed forward primer specific to *lutP* gene, and reverse primers were designed taking the coding regions of *lutR*, *lutD*, and *lutE* genes. In agreement with the *in silico* predictions, which indicated the organization of the *lut* cluster as a single transcriptional unit, we successfully obtained an amplicon of approximately 5.4 kb in a RT-PCR reaction performed using forward and reverse primers, specific to *lutP* and *lutE* regions (Fig. 5*B*, lane 1).

Given the polycistronic nature of *lut* mRNA, we have designed further studies to enhance our comprehension of the impact of *AbcR200* on the translation of *lut* mRNA. As described in the methods sections, we have constructed a broad host range plasmid (pPS5) that carries entire *lut* operon of *A. baumannii* DS002 along with an additional ORF coding mCherry at the intergenic regions of *lutD* and *lutE* genes (Fig. 5*A*). The plasmid, pPS5 borne *lut* operon variant, *lut’* codes mCherry, in addition to *lut* specific proteins from its native promoter identified upstream of *lutP* gene (Fig. 5*A*). The WT DS002 and HS002 strains of *A. baumannii carrying* plasmid, pPS5 were then used to monitor the expression of *lut* operon by measuring the fluorescence. Supporting the *AbcR200* dependent translation inhibition of *lut* operon, the WT DS002 (pPS5) strain emitted less amount of fluorescence (Fig. 5, *C* and *E*) when compared to the amount of fluorescence emitted by the HS002 (pPS5) cells (Fig. 5, *D* and *E*). These findings align with *in silico* studies and increased expression of *lut* genes in HS002 cells of *A. baumannii*, strongly suggests inhibitory role of *AbcR200* on the translation of *lut* mRNA (Fig. 4, *A* and *B*, rows I, II, and III).

**Figure 6:**
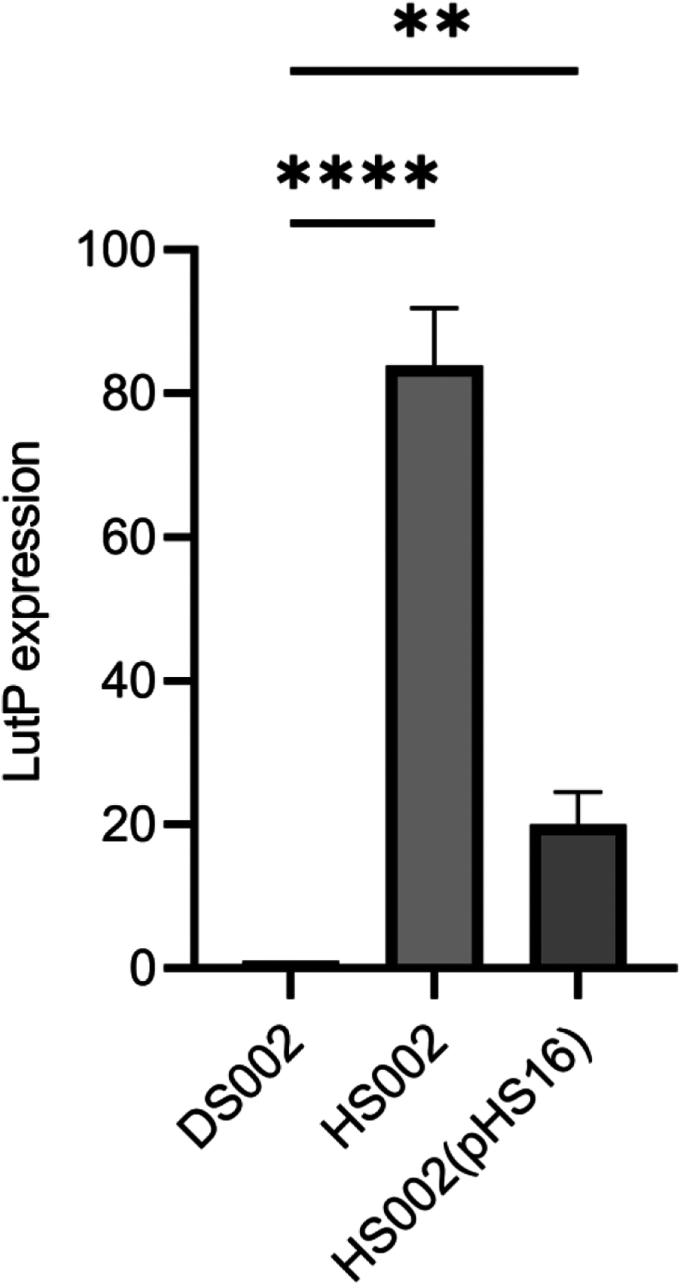
**The *abcR200* negative *A. baumannii* HS002 cells were electroporated with a broad host range plasmid, pHS16 carrying *abcR200* gene.** The total RNA isolated from HS002 (pHS16), HS002, and WT *A. baumannii* DS002 cells was used to quantify *LutP* mRNA by performing qPCR. Graph represents concentration of *LutP* mRNA in DS002, HS002, and HS002 (pHS16) cells. One-way Anova was used to plot the data, and it represents mean ± SD of three independent experiments. Statistical significance of data wherever applicable is indicated by ∗*p* < 0.05; ∗∗∗*p* < 0.001. qPCR, quantitative PCR

As deletion of *abcR200* disrupts *omt* gene, we have created *abcR200* negative and *omt* positive genetic background in HS002 by transforming _*Ab*_Omt^C6His^ coding plasmid, pHS14 and quantified *lut* specific mRNA in the presence of ectopically expressed Omt (Fig. S5). Aligning with the whole body of the data generated in this study, even in the presence of ectopically expressed Omt protein, all *lut* genes were found upregulated in *A. baumannii* HS002 (pHS14) cells (Fig. 4*C* rows I, II, and III). Especially, the *lutP* gene expression has gone up by 35 folds in HS002 (pHS14) cells (Fig. 4*C*, row I). Finally, we have created *abcR200* positive genetic background in HS002 cells by electroporating a broad host range plasmid, pHS16 generated by cloning the *abcR200* gene along with its native promoter. The HS002 (pHS16) strain was then used to quantify the *lutP* specific mRNA. The expression levels of *lutP*-specific transcript got significantly reduced when we created *abcR200* positive background in HS002 cells (Fig. 6). This genetic evidence provides conclusive evidence on *AbcR200* dependent translational inhibition of *lut* operon in *A. baumannii* HS002 cells.

## Discussion

Our investigations have identified Lrp-regulated small RNA, *AbcR200* in *A. baumannii* DS002. Lrp, is a global transcriptional regulator and regulates nearly 40 genes in *E. coli* (58). This dual transcriptional regulator positively regulates genes involved in amino acid biosynthesis, transport, and negatively regulates amino acid degradation (59). In addition to the genes involved in amino acid metabolism, genes coding outer membrane porins, synthesis of pili, aminoacyl tRNA syntheses are also found as part of Lrp regulon in *E. coli* (59). Besides influencing the expression of various genes, the 18 kDa Lrp protein also plays a key role in maintaining the compact structure of the *E. coli* chromosome and regulates its own expression based on the cells nutritional status (59). In nutrient-rich medium, Lrp is made at minimal concentrations, whereas its expression goes up in cells grown in less preferred carbon sources (59). Coinciding with the published results, _*Ab*_Lrp^C6His^ oligomerized in the absence of leucine and interacted with *lrp*-binding motif identified overlapping *abcR200* promoter. Since *abcR200* is part of Lrp regulon, nutritional status of the culture medium has shown significant influence on expression of *abcR200*. Its expression was significantly high in LB grown cultures and showed moderate expression when cells are grown in succinate. Its expression was insignificant in cultures grown using benzoate, probably due to low levels of intracellular leucine concentrations that favors oligomerization of Lrp, the molecular state that makes Lrp as an active repressor. The cross-linking studies done both in presence and absence of leucine clearly supported the proposed hypothesis.

**Figure 7:**
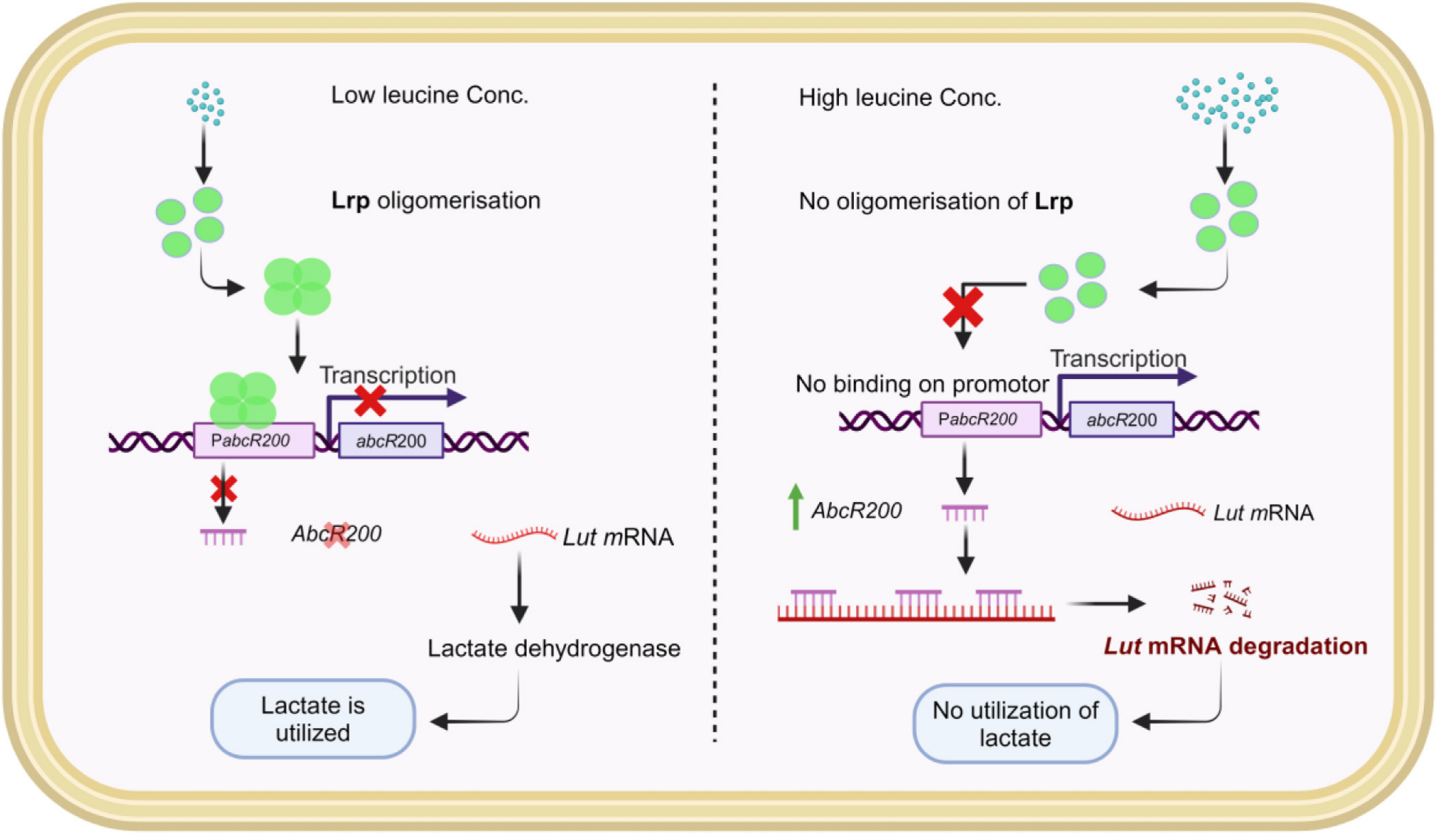
**Model depicting the interplay between *abcR200* expression and regulation of *lut* operon expression in *A. baumannii* DS002.** Low intracellular leucine concentration promotes Lrp oligomerization, a favorable molecular conformation required to repress *abcR200* gene by interacting with P_*abcR200*_. In the absence of *AbcR200*, Lut mRNA is translated to produce lactate dehydrogenase (LDH), enabling *A. baumanni* DS002 to use lactate as source of carbon and energy. Dissociation of Lrp into monomers and dimers in the presence of high intracellular leucine promotes detachment of Lrp from P_*abcR200*_ and promotes derepression of *abcR200* gene. Presence of *AbcR200* prevents translation of *lut* mRNA and promotes its degradation. In the absence of *lut* mRNA translation, LDH is not synthesized, the enzyme required to promote utilization of lactate as source of carbon and energy. Lrp, leucine responsive regulatory protein.

The present study suggests existence of leucine dependent signaling mechanism in sensing carbon status. *A. baumannii* DS002 grows in a variety of ecological niches and frequently encounters fluctuations in carbon source. The cell needs to quickly reorient expression pattern of its catabolic repertoire to facilitate usage of available carbon sources. Under carbon limiting conditions the intracellular amino acid pool serves as a quick source of carbon (60). Particularly, leucine catabolism is favored by the cell as it yields acetyl CoA and acetoacetate that feed directly into tricarboxy (tricarboxylic acidlic acid cycle. Depletion of intracellular leucine concentration, due to its usage as carbon source, has obvious consequences. It serves as a direct chemical signal to indicate carbon starvation and promotes conversion of otherwise inactive Lrp into an active repressor by promoting its oligomerization. The oligomeric Lrp represses expression of *AbcR200*, which has a direct role in translation inhibition of *lut* mRNA. This novel link revolving around leucine, Lrp, and *AbcR200* regulate lactate catabolism in *A. baumannii* DS002 (Fig. 7).

Lactate is used by many pathogenic bacteria as sole source of carbon (61). Ability to use lactate as carbon source directly contributes for enhanced pathogenesis in bacteria like *Neisseria gonorrhoeae*, *Neisseria meningitides*, *S. aureus*, and *Haemophilus influenzae* (62). Pathogens prefer lactate over other sugars as it provides instantaneous energy (63). In a recent study it has been shown that the *A. baumannii* growth increased with parallel increase of lactate concentration in blood plasma (64). The transcriptome analysis done for *A. baumannii* cells isolated from bacteremia mice showed notable increase (7–8 folds) in expression of genes involved in lactate utilization (65). Induction of *lut* operons in *A. baumannii* cells isolated from bacteremia mice has clear link to this study. The *abcR200* gene is conserved among all pathogenic *A. baumannii* strains including the strain isolated from bacteremia mice (Fig. S6). This study clearly established a role for *abcR200* in regulation of *lut* operon. Unless *AbcR200* expression is repressed, increase of *lut*-specific mRNA is not possible in cells isolated from infected mice. Further studies are required to understand the regulation of *abcR200* expression during various stages of infection and to establish its role in pathogenesis. The current study presents experimental evidence linking intracellular leucine concentration and lactate catabolism and reveals yet another adaptive mechanism existing in *A. baumannii* DS002 to counter carbon limiting conditions effectively (Fig. 6).

## Experimental procedures

### Strains and media

Bacterial strains, plasmids, and primers used in this study are listed in main Tables 1 and 2, respectively. All biochemicals and enzymes used in DNA manipulations were procured from Thermo Fisher Scientific India Pvt Ltd, DSS Takara Bio India Pvt Ltd, and Merck India Pvt Ltd. The *A. baumannii* DS002 cells were grown at 30 °C either in LB medium or in minimal medium (275 mM K_2_HPO_4_, 8.8 mM KH_2_PO_4_, 12 mM NH_4_NO_3_, 2 mM MgSO_4_.7H_2_O, 50 μM Fe(SO_4_)_3_, and 0.1 mM CaNO_3_. 4H_2_O) containing succinate (5 mM) or benzoate (5 mM) or benzoate (2.5 mM) + succinate (2.5 mM) or lactate (5 mM) as a sole source of carbon. When required, antibiotics ampicillin (100 μg/ml), kanamycin (30 μg/ml), chloramphenicol (30 μg/ml), and gentamycin (20 μg/ml) were added to the growth medium. The antibiotic concentrations were reduced to half while growing the cultures in lactate containing minimal medium. The filter sterilized leucine (5 mM) was supplemented to the culture medium while assessing its influence on expression of *lut* operon. The *E. coli* strains were grown in LB medium at 37 °C. Routine gene manipulations, Western blots, nucleic acid hybridization techniques, and β-galactosidase assays were performed by following standard protocols (66, 67).

**Table 1:** Strains and plasmids

S. No.	Strain or plasmid	Genotype or phenotype	Reference or source
Strains			
1	*Escherichia coli* DH5α	*λsupE*44, *ΔlacU*169 (*Δ80 lacZΔM*15) *hsdR*17 *recA*1 *endA*1 *gyrA*96 *thi*1 *relA*1	(92)
2	*E. coli* Bl21- DE3	*F – ompT gal dcmlonhsdSB (rB –mB –) λ (DE3 [lacI lacUV5-T7p07 ind1 sam7 nin5]) [malB+]K-12 (λS)*	(93)
3	*E. coli* S17–1	*recAprohsdRRP42Tc::MuKm::Tn7* integrated into the chromosome.	(94)
4	JW0872–2	*F-, Δ (araD-araB) 567, ΔlacZ4787 (::rrnB-3), λ -, Δlrp-787::kan, rph-1, Δ (rhaD-rhaB) 568, hsdR514*	(82)
5	*E. coli* HS001	*K-12 F– λ – ilvG– rfb-50 rph-1Δlac Δlrp*	This study
6	*E. coli* AM001	*K-12 F– λ – ilvG– rfb-50 rph-1, Δlac*	(95)
8	*Acinetobacter baumannii* DS002	Native WT strain. Cm^r^, Sm^r^	(28)
9	*Acinetobacter baumannii* HS002	*Acinetobacter baumannii* DS002 derivative, generated by deleting *abcR200*gene.	This study
Plasmids			
1	pTZ57R/T	Amp^r^, TA vector used for cloning of PCR products.	Thermo Fisher Scientific, USA
2	pMD20	Amp^r^, TA vector used for cloning of PCR products.	Takara Bio, India
3	pMMB206	Cm^r^, low copy, broad host range, mobilizable expression vector. Drives transcription of cloned genes from an IPTG inducible *tac* promoter.	(96)
4	pYH206	Amp^r^, derivative of pMMB206	This study
5	pMP220	Tet^r^, promoter probe vector.	(78)
7	pET23b	Amp^r^. The T7 promoter containing expression vector. Facilitates expression of cloned genes with C-terminal 6xHis-tag.	Novagen
8	pJQ210	Gm^r^, Suicidal vector used for generating*abcR200* knockouts in *A*. *baumannii* DS002.	(83)
9	pUC4KIXX	Km^r^, contains excisable kanamycin cassette.	(97)
10	pHS1	Gm^r^, pJQ210 derivative. Generated by ligating upstream and downstream flanking regions (500 bp) of *abcR200*as *Not*I fragment. The *abcR200* is replaced with an unique *Bam*HI site.	This study
11	pHS2	Gm^r^, Km^r^, and pHS1 derivative. Generated by inserting the kanamycin cassette at the unique *Bam*HI site.	This study
12	pHS3	Amp^r^, expression plasmid generated by cloning *lutR* gene of *A. baumannii* DS002 in pET23b as *Nde*I and *Xho*I fragment. Codes LutR^C6His^.	This study
13	pHS4	Amp^r^, expression plasmid generated by cloning *lutE* gene of *A. baumannii* DS002 in pET23b as *Nde*I and *Xho*I fragment. Codes LutE^C6His^.	This study
14	pHS5	Amp^r^, expression plasmid generated by cloning *lutP* gene of *A. baumannii* DS002 in pET23b as *Nde*I and *Xho*I fragment. Codes LutP^C6His^.	This study
15	pHS6	Amp^r^, pHY206 derivative, generated by cloning *lutR* gene amplified from pHS3 as *Sma*I and *Sal*I fragment. Codes LutR^C6His^.	This study
16	pHS7	Amp^r^, pHY206 derivative, generated by cloning *lutE* gene amplified from pHS4 as *Eco*RI and *Sal*I fragment. Codes LutE^C6His^.	This study
17	pHS8	Amp^r^, pHY206 derivative, generated by cloning *lutP* gene amplified from pHS5 as *Eco*RI and *Sal*I fragment. Codes LutP^C6His^.	This study
18	pHS9	Amp^r^, generated by cloning 5′ RACE productpTZ57R/T.	This study
19	pHS10	Amp^r^, Expression plasmid generated by cloning *lrp* gene of *A. baumannii* DS002 in pET23b as *Nde*I and *Xho*I fragment. Codes Lrp^C6His^.	This study
20	pHS11	Cm^r^, pMMB206 derivative, generated by cloning *lrp* gene amplified from pHS10 as *Eco*RI and *Hin*dIII fragment. Codes for Lrp^C6His^.	This study
21	pHS12	Tet^r^, generated by cloning promoter region of *abcR200* as *Eco*RI-*Pst*I fragment in pMP220.	This study
22	pHS13	Tet^r^, generated by cloning mutant promoter of abcR200 in pMP220 *Eco*RI-*Pst*I fragment.	This study
23	pPS1	Amp^r^, generated by cloning *lut* operon of *A. baumannii* DS002 in pMD20.	This study
24	pPS2	Amp^r^, pPS1 derivative, generated by introducing *Xho*I and *Nhe*I sites in the intergenic region of *lutD* and *lutE* genes of *lut* operon.	This study
25	pPS3	Amp^r^, pPS2 derivative, generated by inserting ORF coding mCherry at the intergenic region of *lutD* and *lutE* genes of *lut* operon as *Xho*I and *Nhe*I fragment.	This study
26	pTSR6K	Km^r^, Shuttle vector constructed during indigenous plasmid of pTS4 of *A. baumannii* DS002	(28)
27	pPS4	Amp^r^, pTSR6K derivative, generated by replacing kanamycin cassette of pTSR6K with *bla* gene. The *bla* gene is inserted as *Xho*I and *Bam*HI fragment.	This study
28	pPS5	Amp^r^, pPS4 derivative, generated by ligating *lut* operon obtained from pPS3 as BamHI and *Sal*I fragment. Contains ORF coding mCherry at the intergenic region of *lutD* and *lutE* genes.	This study
29	pHS15	Amp^r^ generated by cloning *omt* gene as *Nde*I and *Xho*I fragment in pET23b. Codes for Omt^C6His^	This study
30	pHS14	Cm^r^ generated by cloning *omt* gene amplified from pHS15 in pMMB206 as *Eco*RI and *Hin*dIII fragment. Codes for Omt^CFLAG^	This study
31	pHS16	Amp^r^ generated by cloning a*bcR200* in pYH206	This study

**Table 2:** List of primers.

S.No.	Name	Sequence (5′ to 3′)	Description
1	HS1 FP	CGTCAATGTCTGTCCAC ATCTGCTGCATAG	*AbcR200* specific forward primer used while performing real time PCR to quantify the transcript of *abcR200* gene
2	HS1 RP	TCCCCTTATGCGCTTCA GTTTAAGGGGATTTGC TC	*AbcR200* specific reverse primer used while performing real time PCR to quantify the transcript of *abcR200* gene
3	HS3	GACAGGTGTAGACGAC GTA TCAGTTTAAAT	*AbcR200* specific DNA oligonucleotide probe used while performing Northern blot analysis.
4	HS4 FP	ATCCTCTA**GCGGCCGC G**GTTTCACGAATGCCT TCAATA	Forward primer used to amplify *abcR200* upstream region. The *Not*I site appended to facilitate cloning is shown in bold case.
5	HS4 RP	ATCGTCA**GGATCC**TCC CGAAGATCCTCGACTC GAC	Reverse primer used to amplify *abcR200* upstream region. The *Bam*HI site appended to facilitate cloning is shown in bold case.
6	HS5 FP	ATCGCTTA**GGATCC**GA CGTATTGCGGGTTAAG TCAGG	Forward primer used to amplify *abcR200* downstream region. The *Bam*HI site appended to facilitate cloning is shown in bold case.
7	HS5 RP	TACGTCAT**GTCGAC**GG TGGGTTGCGCCAGCTC TCC	Reverse primer used to amplify *abcR200* downstream region. The *Sal*I site appended to facilitate cloning is shown in bold case.
8	HS6 FP	GCGTCAGCGTGTGCTG AATGACATGT	Primers used to quantify transcript of *lutD* gene by performing real time PCR.
9	HS6 RP	CCACATCGGGCGATTA ATTGCAGGTGC	
10	HS7 FP	GTACTGATCAGTCGCC GTGGCGATGG	Primers used to quantify transcript of *lutR* gene by performing real time PCR.
11	HS7 RP	CGTAACGCGGCATACC ATGCAGTC	
12	HS8 FP	ACGCGTCAGTACCGTC AAGGTCGTC	Primers used to quantify transcript of *lutE* gene by performing real time PCR.
13	HS8 RP	GACTTGCTTGCCGTCAT GGATGACTTG	
14	HS9 FP	GGCGATTATGGATGGC TGGCGTGG	Primers used to quantify transcript of *lutP* gene by performing real time PCR.
15	HS9 RP	CAGAGTCTGACCCGCT TCAGGTTC	
16	HS10 FP	CTTAGT**CATATG**ATGA GAATCTCCGATCAAGT G	Forward primer used to amplify *lutR* gene from *A. baumannii* DS002 chromosomal DNA. The *Nde*I site appended to facilitate cloning of *lutR* gene in pET23b vector is shown in bold case
17	HS10 RP	GATAAT**CTCGAG**TTTT GAATCAACTCTATTTA AACGG	Reverse primer used to amplify *lutR* gene from *A. baumannii* DS002 chromosomal DNA. The *Xho*I site appended to facilitate cloning of *lutR* gene in pET23b vector is shown in bold case.
18	HS11 FP	CTATTGCATATGATGC AAACATTTTCTCCTCA AGCAG	Forward primer used to amplify *lutE* gene from *A. baumannii* DS002 chromosomal DNA. The *Nde*I site appended to facilitate cloning of *lutE* gene in pET23b vector is shown in bold case.
19	HS11 RP	GACCAA**CTCGAG**AGAT TTAGCCTTACC	Reverse primer used to amplify *lutE* gene from *A. baumannii* DS002 chromosomal DNA. The *Xho*I site appended to facilitate cloning of *lutE* gene in pET23b vector is shown in bold case.
20	HS12 FP	GAGGTG**CATATG**ATGC TCAATATGTGGCAAC	Forward primer used to amplify *lutP* gene from *A. baumannii* DS002 chromosomal DNA. The *Nde*I site appended to facilitate cloning of *lutP* gene in pET23b vector is shown in bold case.
21	HS12 RP	GACATT**CTCGAG**TGGA ATCATCCACGGAACCA G	Reverse primer used to amplify *lutP* gene from *A. baumannii* DS002 chromosomal DNA. The *Xho*I site appended to facilitate cloning of *lutP* gene in pET23b vector is shown in bold case.
22	HS13 FP	CCTATGGATCCCATTCA AATATGTATCCGCT	Forward primer used to amplify *bla* gene from pGEX vector.
23	HS13 RP	GTCTGACAGGGATCCA TGCTTAATCA	Reverse primer used to amplify *bla* gene from pGEX vector.
24	HS14 FP	AACA**GAATTC**AGGGA GACCACAACGGTTTCC C	Forward primer used to amplify genes cloned in pET23b along with sequence coding C-terminus His-tag. The *Eco*RI site appended to facilitate cloning into pYH206 is shown in bold case.
25	HS15 FP	AACA**CCCGGG**AGGGA GACCACAACGGTTTC	Forward primer used to amplify genes cloned in pET23b along with sequence coding C-terminus His-tag. The *Sma*I site appended to facilitate cloning into pYH206 is shown in bold case.
26	HS15 RP	CCTT**GTCGAC**CTAGTT CCTTGTCGACCTAGTT	Reverse primer used to amplify genes cloned in pET23b along with sequence coding C-terminus His-tag. The *Sal*I site appended to facilitate cloning into pYH206 is shown in bold case.
27	Adapter outer primer	GCUGAUGGCGAUGAA UGAACACUGCGUUUGC UGGCUUUGAUGAAA	Adapter specific forward primer used while performing 5′ RACE.
28	HS31 RP	GTCATAAACGTTTAAA ATACGCCTAT	*abcR200* specific outer reverse primer used while performing 5′RACE.
29	HS32 RP	TTAAATGTGAATCGAT ATATTCGTCAA	*abcR200* specific inner reverse nested primer used while performing 5′RACE.
30	HS33 FP	AGATA**GAATTC**AGCCA TTTCTGCATGAGTATCA	Forward primer used to amplify *abcR200* promoter region. The *Eco*RI site appended to facilitate cloning is shown in bold case.
31	HS33 RP	CATGA**CTGCAG**GCAGC AGATGTGGACAGACA	Reverse primer used to amplify *abcR200* promoter region. The *Pst*I site appended to facilitate cloning is shown in bold case.
32	HS34 FP	GCATAG**GAATTC**GATG ATTTGAAAAGCCGTGT TCGTCGG***GGAAGAGA***A ATGCAAAGTCGAGT	Forward primer used to amplify *abcR200* promoter region with mutations in LRP binding motif. The *Eco*RI site appended to facilitate
			cloning is shown in bold case. The mutated sequence is shown in bold italics.
33	HS34 RP	ACGGAT**CTGCAG**CTCA TTCTATAATCAACCAA	Reverse primer used to amplify *abcR200* promoter region with mutations in LRP binding motif. The *Pst*I site appended to facilitate cloning is shown in bold case.
34	HS35 FP	GAAATCTACGTATGGC GTGGACAGACG	Forward primer used to amplify *lrp* gene from *E. coli* genome.
35	HS35 RP	AAGGCGGCGGCCGCTA CTTAACTTTG	Reverse primer used to amplify *lrp* gene from *E. coli* genome.
36	HS36 FP	CAGGTA**CATATG**CGCC CCTTAGATCGTA	Forward primer used to amplify *lrp* gene from *A. baumannii* chromosomal DNA. The *Nde*I site appended to facilitate cloning of *lrp* gene in pET23b vector is shown in bold case.
37	HS36 RP	GATGTA**CTCGAG**CTTG CTCACATCGAGATATA	Reverse primer used to amplify *lrp* gene from *A. baumannii* chromosomal DNA. The *Xho*I site appended to facilitate cloning of *lrp* gene in pET23b vector is shown in bold case.
38	HS37 RP	CCTT**AAGCTT**CTAGTT ATTGCTCAGCGGTGGC	Reverse primer used to amplify gene cloned in pET23b with C-terminus His-tag. The *Hin*dIII site appended to facilitate cloning into PMMB206 is shown in bold case.
39	PS1 FP	CAAACG GGATCC CGGATATCATAAGCTT TAAAAC	Forward primer used to amplify *lut* operon from *A. baumannii* genomic DNA.
40	PS1 RP	CAAACG CTGCAG AAACCAAAAAGGTATG TTCAG	Reverse primer used to amplify *lut* operon from *A. baumannii* genomic DNA.
41	PS2 FP	AACCTTTTCTCGAGCA AACCGTACTTCCTTGTA CGG	Forward primer used to create *Xho*I restriction site between *lut*D and *lut*E to introduce mCherry between *lut*D and *lut*E of *lut* operon
42	PS2 RP	TACGGTTTGCTCGAGA AAAGGTTTTTGGTTACT TTT	Reverse primer used to create *Xho*I restriction site between *lut*D and *lut*E to introduce mCherry between *lut*D and *lut*E of *lut* operon
43	PS3 FP	TGTACGGTTGCTAGCC AGGCGGCATCCATGCC GCCA	Forward primer used to create *Nhe*I restriction site between *lut*D and *lut*E to introduce mCherry between *lut*D and *lut*E of *lut* operon
44	PS3 RP	TGCCGCCTGGCTAGCA ACCGTACAAGGAAGTA CGGT	Reverse primer used to create *Nhe*I restriction site between *lut*D and *lut*E to introduce mCherry between *lut*D and *lut*E of *lut* operon
45	PS4 FP	ATACCGCTCGAGCATT GTTTTATAAGTGAGAA ATGA	Forward primer used to amplify mCherry for inserting between *lut*D and *lut*E of *lut* operon
46	PS4 RP	ATACTAGCTAGCCTAC TTGTACAGCTCGTCCAT GC	Reverse primer used to amplify mCherry for inserting between *lut*D and *lut*E of *lut* operon
47	PS5 FP	TATCA**CTCGAG**CGCGG AACCCCTATTTG	Forward primer used to amplify *bla* gene from pGEX4T1. The *Xho*I site appended for replacing kanamycin cassette of pTSR6K is shown in bold.
48	PS5 RP	GTGAGT**GGATCC**TTAC CAATGCTTAATCAGT	Reverse primer used to amplify *bla* gene from pGEX4T1. The BamHI site appended for replacing kanamycin cassette of pTSR6K is shown in bold.
49	MET FP	CGC**GAATTC**AACATAC AGGTAAATTTGACTAT GCAGC	Forward primer used to amplify *omt* gene from the genomic DNA of *A. baumannii* DS002
50	MET RP	TACT**AAGCTT**TTACTTGT CGTCATCGTCTTTGTAGTC TTTCACAATTGCAATTG	Reverse primer used to amplify *omt* gene from the genomic DNA of *A. baumannii* DS002
51	ChIP FP	AGCCATTTCTGCATGA GTATCACG	Forward primer used to amplify P*abcR200* promoter region of *abcR200* from IPs (immunoprecipitates) obtained from the lysates of *E. coli* AM001 (pHS12) cells.
52	ChIP RP	GCAGCAGATGTGGACA GACATTGA	Reverse primer used to amplify P*abcR200* from IPs obtained from the lysates of *E. coli* AM001 (pHS12) cells of *A.baumannii* that binds to LRP
53	ChIP-LM- FP	GCATAGGAATTCGATG ATTTGAAAAGCCGTGT TCGTCGGGGAAGAGAA TGCAAAGTCGAGT	Forward primer used to amplify P*abcR200* having mutant Lrp binding site from IPs obtained from the lysates of *E. coli* AM001 (pHS13) cells.
54	ChIP-LM- RP	ACGGATCTGCAGCTCA TTCTATAATCAACCAA	Reverse primer used to amplify P*abcR200* having mutant Lrp binding site from IPs obtained from the lysates of *E. coli* AM001 (pHS13) cells
55	AbcR-FP	CCATGATTAC**GAATTC** CACGTCCTAACCAGAT TGTACTG	Forward primers used to amplify *abcR200* gene from genomic DNA of *A.baumannii to* facilitate infusion cloning
56	AbcR-RP	TTGGCTGCAG**GTCGAC** TACTTGGGGACCTGAC TTAACCCG	Reverse primers used to amplify the *abcR200* gene from genomic DNA of *A.baumannii* to facilitate in-fusion cloning
57	Infusion-FP	GTCGACCTGCAGCCAA GCTTGGCACTG	Forward primers to amplify pYH206 for in- fusion cloning
58	Infusion-RP	GAATTCGTAATCATGG TCATAGCTGTTTCCTG	Reverse primers used to amplify pYH206 for in-fusion cloning

### *In-silico* tools

Prediction of small RNAs in *A. baumannii* DS002 genome was made using both sRNA identification protocol using high-throughput technologies (68) and sRNA scanner (69). The predicted small RNAs were then analyzed to predict prokaryotic promoter elements using Bprom (70). Sequence found 100 bp upstream and 30 bp downstream of the predicted sRNA promoters was then used as input to predict transcription factor binding sites using MEME suite (71). The MEME predicted signature sequences were further validated by TOMTOM to obtain similarity scores by comparing with the transcription factor binding motifs available in the data base (72). The sRNA/mRNA interactions were predicted using IntaRNA 2.0 (57). The potential promoter and terminator elements of sRNA coding genes and *lut* operon were predicted by using web-based tools Bprom (73) and Arnold (74), respectively, using default parameters. Online tool Mfold (75) was used to predict secondary structure of *AbcR200*.

### Expression profiling of small RNAs

Slot blot analysis was done to assess expression profile of *in silico* predicted small RNAs in response to various carbon sources. Oligonucleotides complementary to the predicted sRNA sequences were designed and used as probe (Table 2). The probes were end-labeled following standard procedures, and slot blot experiments were performed as described elsewhere (76). Briefly, the cells were grown to mid-log phase either in LB or in minimal medium containing succinate or benzoate or both succinate and benzoate as sole source of carbon. Total RNA was isolated from these cultures following procedures described elsewhere (77). After assessing the integrity of isolated RNA, equal amounts of RNA (10 μg) were taken to make slots on nitrocellulose membrane cut to the size of the minifold (Bio-Rad Bio-Dot SF, Schleicher and Schuell Minifold II) and used to perform hybridization. While performing Northern blots, the isolated total RNA (40 μg) was separated on a 7M urea polyacrylamide gel (16%), and hybridization was performed following standard protocols using end-labeled oligo (5′-GACAGGTGTAGACGACGTATCAGTTTAAAT-3′) complementary to *abcR200* (76).

### Determination of TSS

Briefly, 5′ RACE experiments were performed to determine TSS of *abcR200* gene. The 5′ RACE was performed using First Choice RLM-RACE Kit (Ambion Life technologies) following manufacturers protocol. RNA was isolated from *A. baumannii* DS002 cells grown to late log phase (absorbance of 1) in LB medium. After establishing RNA integrity, 5 μg of DNase-treated RNA was taken in sterile eppendorf tube and treated with tobacco acid pyrophosphatase to remove pyrophosphate from full-length mRNA molecules. Following removal of pyrophosphate, 45 mer RNA adapter oligonucleotide was ligated by incubating the tobacco acid pyrophosphatase-treated RNA at 37 °C for 1 h in presence of T4 RNA ligase. The adapter ligated RNA was then used as template for first strand cDNA synthesis. The cDNA thus synthesized was used to amplify the 5′ end of *abcR200*. A nested PCR was performed using adapter specific outer forward primer and gene specific reverse primers (HS31RP and HS32RP). The amplicon obtained was cloned in T-vector. The recombinant plasmid, pHS9 was used to determine sequence of RACE product and to establish transcription start point of *abcR200*.

### Construction of *abcR200-lacZ* transcriptional fusions

The promoter fusions of *abcR200* were generated by cloning the predicted promoter along with the Lrp-binding site in promoter test vector pMP220 (78). The *abcR200* promoter region was amplified from genomic DNA of *A. baumannii* DS002 using primer set (HS33FP/HS33RP) appended with *Eco*RI and *Pst*I restriction sites. The amplicon was gel extracted and digested with *Eco*RI and *Pst*I before ligating it into pMP220 digested with similar enzymes. The resulting promoter fusion (*abcR-lacZ*) construct was named as pHS12. Promoter sequence with mutations at LRP binding site was amplified using primer set HS34FP/HS34RP. The forward primer HS34FP was designed by introducing mutations in the consensus LRP binding site (TATTTTTT). The conserved (TATTTTTT) bases found in the LRP binding site were changed to GGAAGAGA. While introducing these changes, the −10 and −35 hexamers were left unaltered. The amplicon obtained was sequenced to confirm the presence of mutations in LRP binding site before cloning it into promoter probe vector. The resulting *abcR-lacZ* with mutated LRP binding site was named as pHS13.

### Insertion of ORF coding mCherry at the intergenic region of *lutD* and *lutE*

To clone ORF coding mCherry at the intergenic region of *lutD* and *lutE*, entire *lut* operon including promoter elements was amplified using PS1FP/PS1RP primer set and cloned in T-vector, pMD20. The resulting recombinant plasmid, pPS1 was then used as template to engineer *Xho*I and *Nhe*I sites at the intergenic region of *lutD* and *lutE*. Site-directed mutagenesis was performed by overlap extension PCR (79) using Phusion polymerase (Thermo Fisher Scientific India Pvt Ltd). Initial round of mutagenesis was performed by using pPS1 as template and PS2FP/PS2RP as primers. After ascertaining the creation of *Xho*I site, the second round of site-directed mutagenesis was performed using primer set PS3FP/PS3RP to create *Nhe*I site downstream of *Xho*I site. The resulting plasmid designated as pPS2 was used to insert ORF coding mCherry as *Xho*I and *Nhe*I fragment, the resulting plasmid pPS3 contains entire *lut* operon along with ORF coding for mCherry between *lutD* and *lutE*. The mCherry coding *lut* operon was then cloned in shuttle vector pPS4 as *Bam*HI and *Sal*I fragment. The generated plasmid pPS5 was then electroporated into *abcR200* positive (DS002) and negative (HS002) strains of *Acinetobacter baumanii*.

### Confocal microscopy

*A. baumannii* DS002 (pPS5) and HS002 (pPS5) cells were grown in minimal medium having 5 mM leucine and lactate as sole source of carbon and harvested before mid-log phase. The cells were washed in PBS buffer and resuspended in the same buffer to obtain final *A*_600_ of 5. A 10 μl drop was sealed with a cover slip on a glass slide. The cells were then observed under the super resolution STED microscope (Leica) (laser power-2%, HYD gain-100, PMT-450, objective-100X, and zoom factor-1.90).

### Expression and purification of _*Ab*_LRP^C6His^

*E coli* BL21 (pHS10) cells were grown to mid-log phase and the expression of _*Ab*_LRP^C6His^ was induced following standard procedures (67). After induction, the cells were lysed, and the clear lysate was obtained after centrifugation at 13,000 rpm. The lysate was then used to detect presence of _*Ab*_LRP^C6His^ by performing Western blot using anti-His antibodies. The _*Ab*_LRP^C6His^ found in the lysate was purified using metal ion affinity chromatography standardized in our laboratory (80) and the purity of _*Ab*_LRP^C6His^ was ascertained by analyzing the purified protein on SDS-PAGE (12. 5%).

### Generation of *lrp* null mutant of *E. coli* AM001

*lrp* gene from *E.coli* AM001 was deleted following established procedures (81). Phage particles obtained after infecting *lrp* keio mutant of *E.coli* (82) were used to delete *lrp* gene from *E.coli* AM001. Colonies obtained after transduction were tested for deletion of *lrp* gene by performing colony PCR using the oligos HS35FP and HS35RP as primers. The *lrp* deletion derivative of *E. coli* AM001 strain was named as *E. coli* HS001.

### LRP-*abcR200* interactions: Two plasmid assay

A two-plasmid assay was developed to assess the role of Lrp on expression of *abcR200*. The *E. coli* HS001 strain was cotransformed with pHS12 (*abcR200*–*lacZ* fusion) and pHS11 coding _*Ab*_Lrp^C6His^ from an inducible promoter. The *E. coli* HS001 (pHS11+ pHS12) cells were then used to determine _*Ab*_Lrp^C6His^ dependent repression on the expression of *abcR200*. The *E. coli* HS001 (pHS11+ pHS12) cells were grown in the presence of IPTG to induce the expression of _*Ab*_Lrp^C6His^, and the promoter activity of *abcR200* was assayed by measuring the β-galactosidase activity following the standard procedures (66). The *E. coli* HS001 containing either pHS12 or pHS11 along with *E. coli* HS001 (pHS11+ pHS12) cells grown in the absence of IPTG served as controls. While assessing the influence of leucine on *abcR200* promoter activity, the *E. coli* HS001 (pHS11+ pHS12) cells were grown in minimal medium containing 0. 5, 1.0, 1.5, and 2.0 mM of exogenous leucine and without leucine. When the culture reached to mid-log phase, the cells were harvested and β-galactosidase activity was measured following standard procedures (66). *E. coli* HS001 (pHS11+ pHS12) cells grown in the absence of exogenous leucine served as control culture. Results of three independent experiments were used to perform statistical analysis using GraphPad Prism software. Data are presented as mean ± SD, and the *p* value of < 0.05 was considered statistically significant.

### Leucine induced oligomerization of _*Ab*_LRP^C6His^

The affinity purified _*Ab*_LRP^C6His^ was dialyzed against 20 mM Hepes, 150 mM NaCl, pH 8.0 buffer for 12 h. After dialysis 23 μM protein was taken in a clean eppendorf tube and the process of cross-linking was initiated by adding 1.2 μl (3 mM) DSS (Thermo Fisher Scientific) dissolved in dimethyl sulfoxide. The reaction mix was incubated at room temperature for 30 min to ensure good cross-linking among LRP proteins. When required either leucine (5 mM) or DNA (300 ng) containing LRP binding site was added to the reaction mix. The cross-linking reaction was stopped by the addition of quenching solution (1M Tris–Cl, pH-7.5) to a final concentration of 20 mM. Pure protein without any cross-linker served as control. These samples were analyzed on SDS-PAGE (12.5%), and Western blots were performed using anti-His antibodies to detect the oligomeric state of _*Ab*_Lrp^C6His^. The His-tagged protein mix loaded along with the samples served as molecular weight markers.

### Generation of *abcR200* KOs

The *abcR200* was deleted in *A. baumannii* DS002 by replacing it with kanamycin cassette. As illustrated, the *abcR200* gene overlaps with the *omt* gene and is transcribed in the opposite direction (Fig. 1*A*). The promoter of *omt* gene is in the middle of *abcR200* gene and hence its deletion invariably disrupts *omt* gene promoter region. Briefly, upstream and downstream regions (500 bps) flanking *abcR200* gene were amplified as *Not*I-*Bam*HI and *Bam*HI–*Sal*I fragments using primers HS4FP/HS4RP and HS5FP/HS5RP, respectively. The purified PCR products were then digested with *Bam*HI and ligated to generate a single *Not*I-*Sal*I fragment. The kanamycin resistance cassette was then inserted at *Bam*HI site before ligating it as *Not*I and *Sal*I fragment into a suicidal vector (pJQ210) digested with similar enzymes (83). The resulting recombinant plasmid, pHS2 was used to transform *E. coli* S17-1. Biparental mating using *E. coli* S17-1 (pHS2) as a donor and *A. baumannii* DS002 as recipient was performed following standard procedures, and selection of exconjugants was done on LB plates containing kanamycin (84). The exconjugants were then screened for sucrose sensitivity (5% sucrose plates), and the colonies that lost vector backbone were selected to perform colony PCR using primer set HS4FP and HS5RP. The colony that contained kanamycin cassette in place of *abcR200* gene was designated as *A. baumannii* HS002.

### Transcriptomics

Transcriptome analysis of *A. baumannii* DS002 and *abcR200* deletion derivative, *A. baumannii* HS002 was performed to identify the *abcR200* responsive mRNAs. Three biological replicates of *A. baumannii* DS002 and *A. baumannii* HS002 were grown in carbon rich (LB) medium under identical conditions till late log phase. The cells were harvested, and the total RNA was isolated following the procedures mentioned elsewhere (85). Prior to library preparation, ∼2.5 μg of total RNA was depleted of rRNA using Ribo-Zero rRNA Removal Kit (Bacteria). About ∼50 ng of Qubit quantified ribo-depleted RNA was taken for fragmentation and priming. The fragmented and primed mRNA was further subjected to first strand synthesis in the presence of actinomycin D (Gibco, Life Technologies) followed by second strand synthesis. The double stranded cDNA was purified using HighPrep PCR magnetic beads (MAGBIO Genomics Inc). The purified cDNA was end-repaired, adenylated, and ligated to Illumina multiplex barcode adapters as per NEBNext Ultra Directional RNA Library Prep Kit protocol. The adapter ligated cDNA was purified using HighPrep beads and subjected to 14 cycles of indexing PCR (37 °C) for 15 min followed by denaturation at 98 °C for 30 s, and cycling (98 °C for 10 s, 65 °C for 75 s, and 65 °C for 5 min) to enrich the adapter-ligated fragments. The final PCR product (sequencing library) was purified with HighPrep beads and was subjected to library quality control check. The Illumina-compatible sequencing library was initially quantified by Qubit fluorometer (Thermo Fisher Scientific) and its fragment size distribution was analyzed on Agilent TapeStation. The libraries generated were subjected to Illumina sequencing. Raw sequencing reads were processed to remove the adapter sequences and the low-quality bases using Trimmomatic (v0.38) (http://www.usadellab.org/cms/?page=trimmomatic) (86) software with the following settings in paired end mode. LEADING/TRAILING:10 (1% error), SLIDINGWINDOW:5:16 (window size: quality ∼ 2.5% error), MINLEN:30. FastQC program was used to check read quality before and after the data cleaning (https://www.bioinformatics.babraham.ac.uk/projects/fastqc/). Complete read count table is provided in the Table S1. Data cleaning and analysis of differential expression was performed on a cloud genomics platform (Stanome Pvt. Ltd).

### Differential gene expression analysis

Reference genome and annotation (Genbank ID: CP027704.1) was downloaded from Genbank. Associated gff3 file with all the gene features was also obtained from the same source. Read abundance quantification was performed using salmon tool (v 0.11.2) (87). Normalized and filtered read counts were used for identifying the DEGs using the DESeq2 (88) tool with default settings. DEGs with a q-value of ≤ 0.10 and a log2 fold-change of ≥ 1 (upregulated) or log2 fold-change of ≤ 1 (downregulated) were selected as significant DEGs for further analysis. A logarithmic plot was generated to visualize the differentially expressed genes based on the expression pattern and set cutoff value for significance (Fig. S1).

### RT PCR

Total RNA was isolated from bacterial cells using TRIzol Reagent (Sigma-Aldrich) following the manufacturer’s protocol. The concentration of the RNA isolated was determined spectrophotometrically using a Nanodrop. Total RNA isolated was converted into cDNA using Verso cDNA synthesis kit (Thermo Fisher Scientific) following the manufacturer’s protocol. The reactions were stored in −20 °C until further use. RT- PCR was performed using *lutP* specific forward (HS12 FP) and *lutE* (HS11 RP), *lutD* (HS7 RP), and *lutR* (HS10 RP) specific reverse primers to verify if the *lut* genes are organized as an operon in the genome of *A. baumannii* DS002. The RT-PCR was performed following established protocols (89).

### Quantitative PCR

Quantitative PCR (qPCR) was performed to quantify the expression of *lut*-specific mRNAs following standard procedures. The 16S rRNA gene served as an internal control for calibrating the expression of other genes. The qPCR reactions were carried out in Applied Biosystems 7500 Real-Time PCR System operating with ABI 7500 software (https://www.thermofisher.com/mx/es/home/technical-resources/software-downloads/applied-biosystems-7500-real-time-pcr-system.html) using 20 ng of cDNA template, 0.25 μM primers, and 1X Takara SYBR Green master mix. The real-time expression analysis of each gene was carried out in triplicates, and the relative expression of genes were calculated using 2-ΔCt method (90). The expression levels of *lut*-specific mRNAs were quantified following standard procedures. Since deletion of *abcR200*, disrupted *omt* gene, we have complemented HS002 strain with ectopically expressed O-methyltransferase before isolating total RNA. The *A. baumannii* DS002 and HS002 (pHS14) cultures were grown to mid-log phase in a medium containing lactate as sole source of carbon. The total RNA isolated from these cultures was used to quantify the expression levels of *lutD*, *lutR*, *lutE*, and *lutP* genes by performing qPCR.

### Quantification of *lutP* expression in *A. baumannii* HS002 (pHS16)

Initially, the *abcR200* gene along with its indigenous promoter was amplified and ligated in pYH206 to generate recombinant plasmid pHS16 using infusion PCR cloning kit. The plasmid pHS16 was then electroporated into *A. baumannii* HS002 cells to generate *abcR200* positive genetic background. The *A. baumannii* HS002 (pHS16) cells were then grown to mid-log phase in minimal media and used for isolating total RNA. The *lutP* specific transcript was then quantified by performing qPCR by following procedures described elsewhere (90).

### Construction of expression plasmids

The *lutR*, *lutE*, *and lutP* genes were amplified as *Nde*I and *Xho*I fragments using primer pair showed in Table 2 and independently ligated in pET23b. The recombinant plasmids designated as pHS3, pHS4, and pHS5, code LutR^C6His^, LutE^C6His^, LutP^C6His^, respectively. These recombinant plasmids were then transformed into BL21, and their expression was induced following standard procedures. After confirming their expression in *E. coli*, the plasmid pHS3 was used as a template to amplify *lutR* as *Sma*I and *Sal*I fragment and ligated in pHY206 digested with similar enzymes, and the resulting recombinant plasmid was designated as pHS6. Similarly, plasmids pHS4 and pHS5 served as a template to amplify *lutE and lutP* genes as *Eco*RI and *Sal*I fragments. These fragments were then ligated independently in pHY206 to generate broad host range expression plasmids pHS7 and pHS8. These (pHS6, pHS7, and pHS8) plasmids were then independently mobilized into *A. baumannii* DS002 and HS002 strains and ectopically expressed LutR^C6His^, LutE^C6His^, and LutP^C6His^ were monitored by performing Western blots using anti-His antibodies. Similar strategy was followed while expressing *omt* gene in *A. baumannii* HS002 strain. The *omt* gene cloned in pHY206 as *Eco*RI fragment generated broad host range expression plasmid pHS14 and it codes for Omt^C6His^. After checking its expression in *E. coli*, the plasmid pHS14 was mobilized into *A. baumannii* HS002. Expression of Omt in HS002 (pHS14) was induced by adding IPTG (1 mM) to the cultures grown to mid-log phase and its expression was monitored by performing Western blots using anti-His antibodies.

### ChIP assay

ChIP analysis was performed using *lrp* negative *E.coli* HS001 cells. Initially, the *E. coli* HS001 cells were transformed with two compatible plasmids. One of them (pHS11) codes _*Ab*_Lrp^C6His^ from an IPTG inducible promoter and the second plasmid (pHS12) carries *abcR200-lacZ* fusion. Control *E. coli* HS001 cells were generated either by replacing _*Ab*_Lrp^C6His^ coding expression plasmid with expression vector, pMMB206 or by introducing *abcR200-lacZ* fusion (pHS13) with a mutant Lrp binding site. The cultures were grown to mid-log phase either in leucine-free minimal medium or in a minimal medium supplemented with 2 mM and 5 mM leucine before inducing the expression of _*Ab*_Lrp^C6His^ by adding 1 mM IPTG. After inducing the expression of _*Ab*_Lrp^C6His^, the ChIP assays were performed by following the procedure described elsewhere (91). Briefly, cells were incubated with 1% formaldehyde for 10 min at 37 °C to facilitate crosslinking between interacting _*Ab*_Lrp^C6His^ and P_*abcR200*_. After crosslinking, the cells were lysed by adding the lysis buffer (1% SDS+ 10 mM EDTA+50 mM Tris, pH 8.1) and briefly sonicated to facilitate fragmentation of DNA. The cross-linked lysates were then diluted using dilution buffer (0.01% SDS+ 1.2 mM EDTA+ 16.7 mM Tris, pH 8.1 + 167 mM NaCl) and immunoprecipitated overnight at 4 °C using either anti-His or anti-IgG antibodies. Next, Protein A/G beads were added to the immunoprecipitated complex, then placed on shaker and incubated at 4 °C with mild shaking. After incubation, the beads were thoroughly washed with different wash buffers (low salt buffer, high salt buffer, and LiCl buffer) to remove nonspecifically bound antigens to protein A/G beads. The antibody-bound DNA-protein complexes were then eluted using elution buffer (1% SDS + 0.1 M sodium bicarbonate) and incubated at 65 °C for 4 h after adding 5 M NaCl to remove cross-links formed between protein and DNA. After de-cross-linking is completed, the samples were treated with proteinase K, and the degraded proteins were removed by performing phenol–chloroform extraction. Finally, the DNA present in the aqueous phase was precipitated and used as template to amplify P_*abcR200*_ using specific primers listed in Table 2.

## Data availability

All the data presented in this document can be found within the manuscript and accompanying supplementary files.

## Supporting information

This article contains supporting information.

## Conflict of interest

The authors declare that they have no conflicts of interest with the contents of this article.
