# Supplementary material for: A leucine responsive small RNA AbcR200 regulates expression of the lactate utilization (lut) operon in Acinetobacter baumannii DS002
Source: J Biol Chem. 2025 Jan 10;301(2):108160. doi: 10.1016/j.jbc.2025.108160 (PMC11869524; doi:10.1016/j.jbc.2025.108160)
Supplement: Supplementary information [file mmc1.pdf]

Supplementary Figure S1: Carbon responsive small RNA

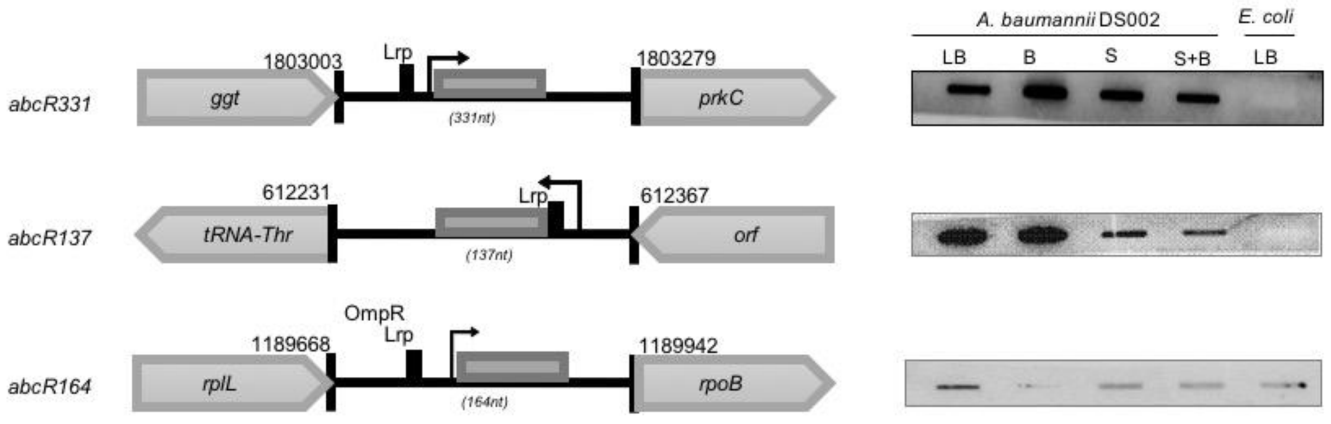

Supplementary Figure S2 : Coomassie gel showing purified lrp

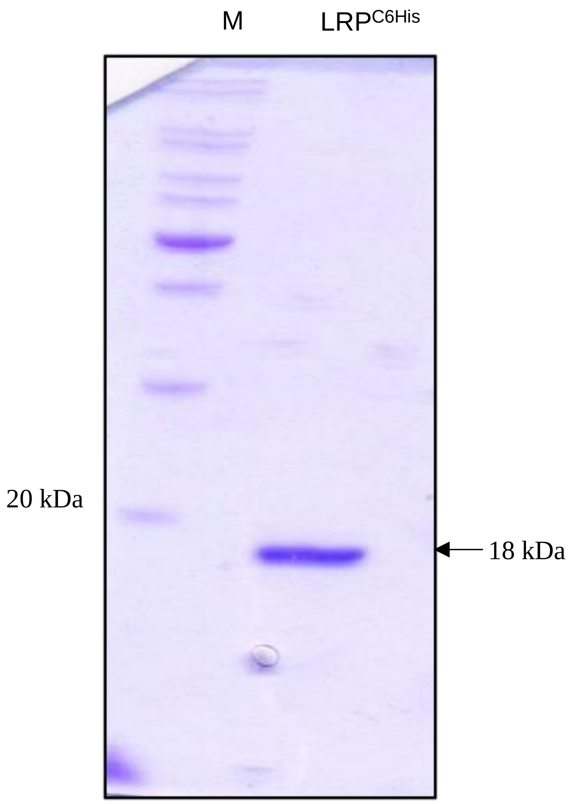

Supplementary Figure S3: ChIP assay

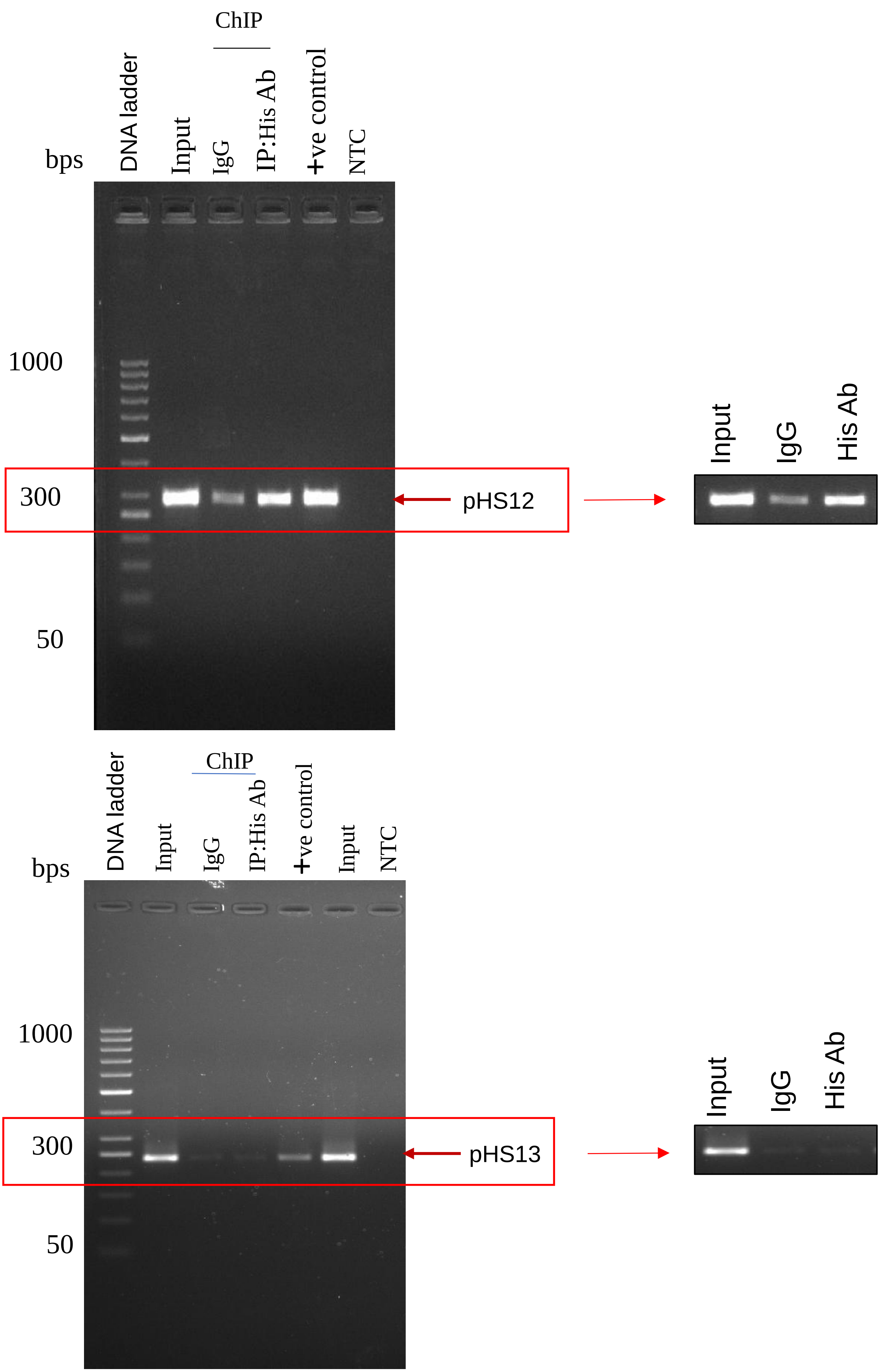

Supplementary Figure S3: ChIP assay

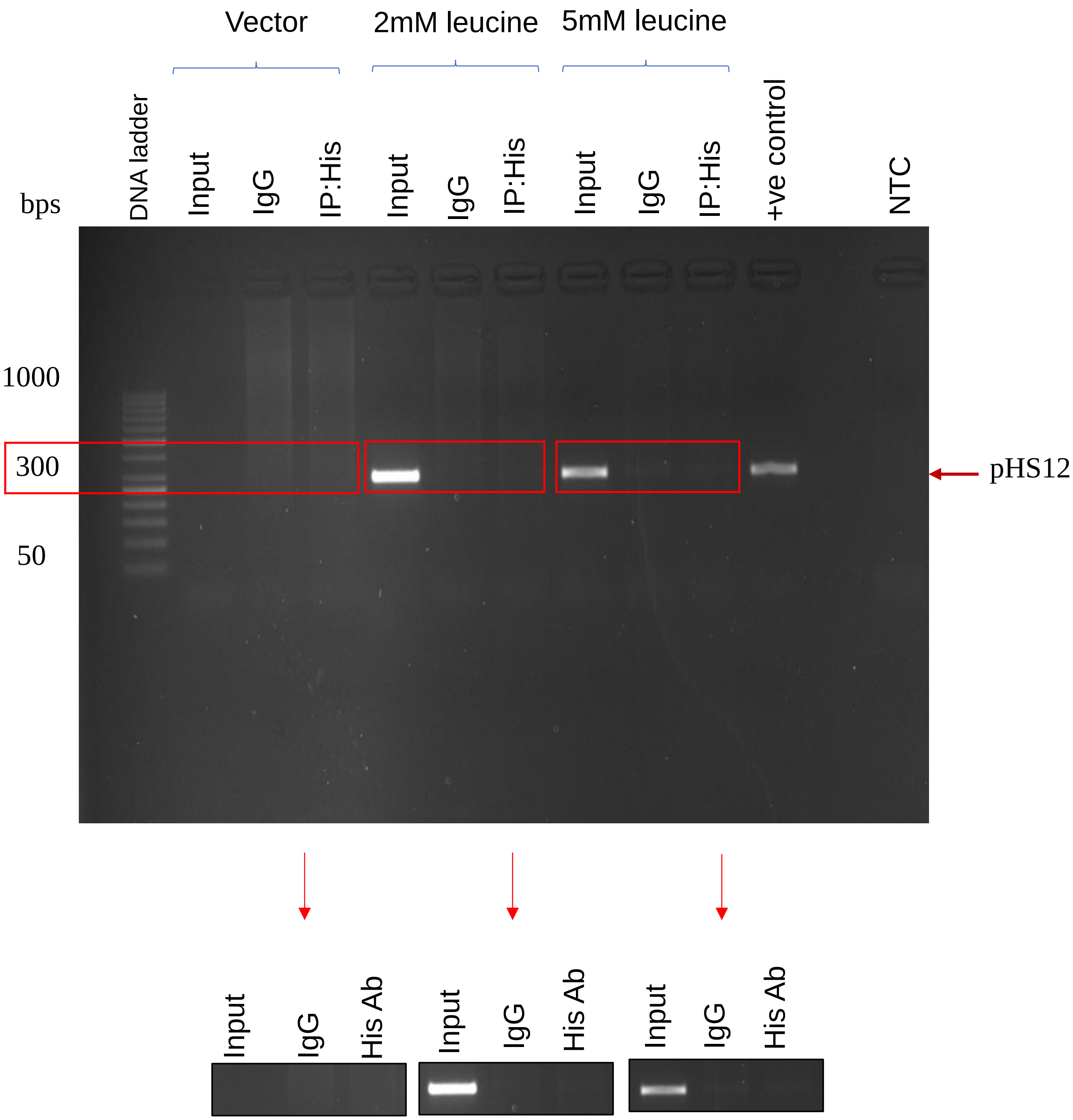

Supplementary Figure S4: Graphical representation of differentially expressed gene in *A. baumannii* HS002

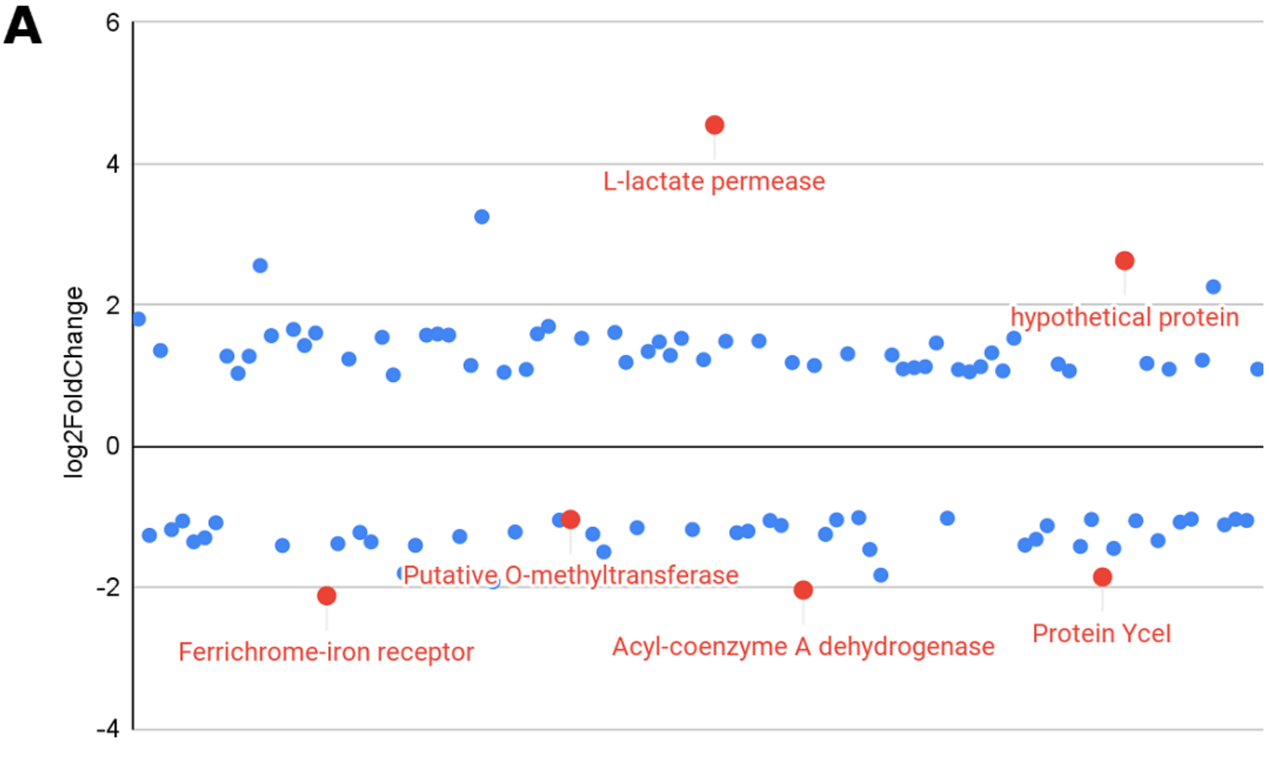

Supplementary Figure S5 : omt gene (pMMB206-omt^{CFLAG}) expression in *E.coli*

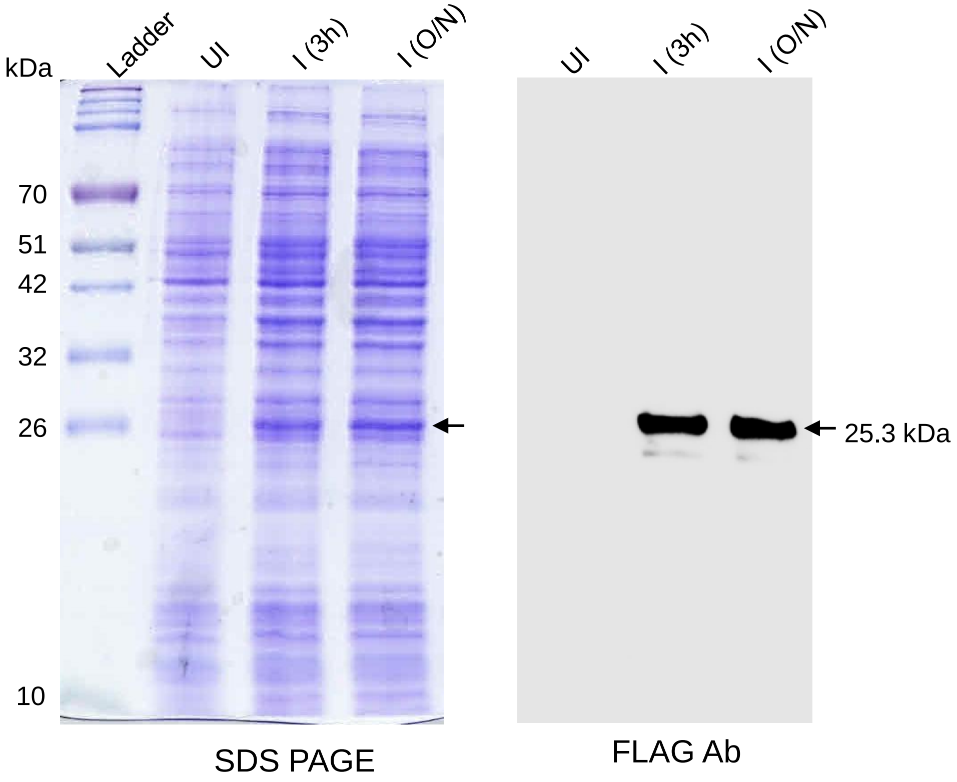

Supplementary Figure S6: Conservation of *abcR200* among different *A. baumannii* strains

```
AbDS002        GCCGTGTTCGTCGGTATTTTTTAATGCAAAGTCGAGTCGAGGATCTTCGGGAATTAAATGTGAAT  65
AbATCC17978    GCCGTGTTCGTCGGTATTTTTTAATGCAAAGTCGAGTCGAGGATCTTCGGGAATTAAATGTGAAT  65
AbXDR-BJ83     GCCGTGTTCGTCGGTATTTTTTAATGCAAAGTCGAGTCGAGGATCTTCGGGAATTAAATGTGAAT  65
AbAYE          GCCGTGTTCGTCGGTATTTTTTAATGCAAAGTCGAGTCGAGGATCTTCGGGAATTAAATGTGAAT  65
AbHWBA8        GCCGTGTTCGTCGGTATTTTTTAATGCAAAGTCGAGTCGAGGATCTTCGGGAATTAAATGTGAAT  65
AbA1           GCCGTGTTCGTCGGTATTTTTTAATGCAAAGTCGAGTCGAGGATCTTCGGGAATTAAATGTGAAT  65
AbD46          GCCGTGTTCGTCGGTATTTTTTAATGCAAAGTCGAGTCGAGGATCTTCGGGAATTAAATGTGAAT  65
AbACICU        GCCGTGTTCGTCGGTATTTTTTAATGCAAAGTCGAGTCGAGGATCTTCGGGAATTAAATGTGAAT  65
AbDT0544C      GCCGTGTTCGTCGGTATTTTTTAATGCAAAGTCGAGTCGAGGATCTTCGGGAATTAAATGTGAAT  65
AbUPAB1        GCCGTGTTCGTCGGTATTTTTTAATGCAAAGTCGAGTCGAGGATCTTCGGGAATTAAATGTGAAT  65
AbLAC-4        GCCGTGTTCGTCGGTATTTTTTAATGCAAAGTCGAGTCGAGGATCTTCGGGAATTAAATGTGAAT  65
AbATCCBAA1605  GCCGTGTTCGTCGGTATTTTTTAATGCAAAGTCGAGTCGAGGATCTTCGGGAATTAAATGTGAAT  65
AbAB5075-VUB   GCCGTGTTCGTCGGTATTTTTTAATGCAAAGTCGAGTCGAGGATCTTCGGGAATTAAATGTGAAT  65
AbMS14413      GCCGTGTTCGTCGGTATTTTTTAATGCAAAGTCGAGTCGAGGATCTTCGGGAATTAAATGTGAAT  65
AbABF9692      GCCGTGTTCGTCGGTATTTTTTAATGCAAAGTCGAGTCGAGGATCTTCGGGAATTAAATGTGAAT  65
AbDT-Ab003     GCCGTGTTCGTCGGTATTTTTTAATGCAAAGTCGAGTCGAGGATCTTCGGGAATTAAATGTGAAT  65

AbDS002        CGATATATTCGTCAATGTCTGTCCACATCTGCTGCATAGTCAAATTTACCTGTATGTTGATAGGC 130
AbATCC17978    CGATATATTCGTCAATGTCTGTCCACATCTGCTGCATAGTCAAATTTACCTGTATGTTGATAGGC 130
AbXDR-BJ83     CGATATATTCGTCAATGTCTGTCCACATCTGCTGCATAGTCAAATTTACCTGTATGTTGATAGGC 130
AbAYE          CGATATATTCGTCAATGTCTGTCCACATCTGCTGCATAGTCAAATTTACCTGTATATTGATAGGC 130
AbHWBA8        CGATATATTCGTCAATGTCTGTCCACATCTGCTGCATAGTCAAATTTACCTGTATGTTGATATGC 130
AbA1           CGATATATTCGTCAATGTCTGTCCACATCTGCTGCATAGTCAAATTTACCTGTATATTGATAGGC 130
AbD46          CGATATATTCGTCAATGTCTGTCCACATCTGCTGCATAGTCAAATTTACCTGTATGTTGATATGC 130
AbACICU        CGATATATTCGTCAATGTCTGTCCACATCTGCTGCATAGTCAAATTTACCTGTATGTTGATAGGC 130
AbDT0544C      CGATATATTCGTCAATGTCTGTCCACATCTGCTGCATAGTCAAATTTACCTGTATGTTGATAGGC 130
AbUPAB1        CGATATATTCGTCAATGTCTGTCCACATCTGCTGCATAGTCAAATTTACCTGTATGTTGATATGC 130
AbLAC-4        CGATATATTCGTCAATGTCTGTCCACATCTGCTGCATAGTCAAATTTACCTGTATATTGATAGGC 130
AbATCCBAA1605  CGATATATTCGTCAATGTCTGTCCACATCTGCTGCATAGTCAAATTTACCTGTATATTGATAGGC 130
AbAB5075-VUB   CGATATATTCGTCAATGTCTGTCCACATCTGCTGCATAGTCAAATTTACCTGTATATTGATAGGC 130
AbMS14413      CGATATATTCGTCAATGTCTGTCCACATCTGCTGCATAGTCAAATTTACCTGTATGTTGATAGGC 130
AbABF9692      CGATATATTCGTCAATGTCTGTCCACATCTGCTGCATAGTCAAATTTACCTGTATGTTGATAGGC 130
AbDT-Ab003     CGATATATTCGTCAATGTCTGTCCACATCTGCTGCATAGTCAAATTTACCTGTATGTTGATAGGC 130

AbDS002        GTATTTTAAACGTTTATGACTAGAGTGCAAAAATCTTCGATTCAAGAAATTTTGGTTGATTATGG 195
AbATCC17978    GTATTTTAAACGTTTATGACTAGAGTGCAAAAATCTTCGATTCAAGAAATTTTGGTTGATTATAG 195
AbXDR-BJ83     GTATTTTAAACGTTTATGACTAGAGTGCAAAAATCTTCGATTCAAGAAATTTTGGTTGATTATAG 195
AbAYE          GTATTTTAAACGTTTATGACTAGAGTGCAAAAATCTTCGATTCAAGAAATTTTGGTTGATTATAG 195
AbHWBA8        GTATTTTAAACGTTTATGACTAGAGTGCAAAAATCTTCGATTCAAGAAATTTTGGTTGATTATAG 195
AbA1           GTATTTTAAACGTTTATGACTAGAGTGCAAAAATCTTCGATTCAAGAAATTTTGGTTGATTATAG 195
AbD46          GTATTTTAAACGTTTATGACTAGAGTGCAAAAATCTTCGATTCAAGAAATTTTGGTTGATTATAG 195
AbACICU        GTATTTTAAACGTTTATGACTAGAGTGCAAAAATCTTCGATTCAAGAAATTTTGGTTGATTATAG 195
AbDT0544C      GTATTTTAAACGTTTATGACTAGAGTGCAAAAATCTTCGATTCAAGAAATTTTGGTTGATTATAG 195
AbUPAB1        GTATTTTAAACGTTTATGACTAGAGTGCAAAAATCTTCGATTCAAGAAATTTTGGTTGATTATAG 195
AbLAC-4        GTATTTTAAACGTTTATGACTAGAGTGCAAAAATCTTCGATTCAAGAAATTTTGGTTGATTATAG 195
AbATCCBAA1605  GTATTTTAAACGTTTATGACTAGAGTGCAAAAATCTTCGATTCAAGAAATTTTGGTTGATTATAG 195
AbAB5075-VUB   GTATTTTAAACGTTTATGACTAGAGTGCAAAAATCTTCGATTCAAGAAATTTTGGTTGATTATAG 195
AbMS14413      GTATTTTAAACGTTTATGACTAGAGTGCAAAAATCTTCGATTCAAGAAATTTTGGTTGATTATAG 195
AbABF9692      GTATTTTAAACGTTTATGACTAGAGTGCAAAAATCTTCGATTCAAGAAATTTTGGTTGATTATAG 195
AbDT-Ab003     GTATTTTAAACGTTTATGACTAGAGTGCAAAAATCTTCGATTCAAGAAATTTTGGTTGATTATAG 195

AbDS002        AATGAGCAAATCCCCTTAAACTGAAGCGCATAAGGGGATTTATATTT                   242
AbATCC17978    AATGAGCAAATCCCCTTAAACTGAAGCGCATAAGGGGATTTATATTT                   242
AbXDR-BJ83     AATGAGCAAATCCCCTTAAACTGAAGCGCATAAGGGGATTTATATTT                   242
AbAYE          AATGAGCAAATCCCCTTAAACTGAAGCGCATAAGGGGATATATATTT                   242
AbHWBA8        AATGAGCAAATCCCCTTAAACTGAAGCGCATAAGGGGATTTATATTT                   242
AbA1           AATGAGCAAATCCCCTTAAACTGAAGCGCATAAGGGGATATATATTT                   242
AbD46          AATGAGCAAATCCCCTTAAACTGAAGCGCATAAGGGGATTTATATTT                   242
AbACICU        AATGAGCAAATCCCCTTAAACTGAAGCGCATAAGGGGATTTATATTT                   242
AbDT0544C      AATGAGCAAATCCCCTTAAACTGAAGCGCATAAGGGGATTTATATTT                   242
AbUPAB1        AATGAGCAAATCCCCTTAAACTGAAGCGCATAAGGGGATTTATATTT                   242
AbLAC-4        AATGAGCAAATCCCCTTAAACTGAAGCGCATAAGGGGATATATATTT                   242
AbATCCBAA1605  AATGAGCAAATCCCCTTAAACTGAAGCGCATAAGGGGATATATATTT                   242
AbAB5075-VUB   AATGAGCAAATCCCCTTAAACTGAAGCGCATAAGGGGATATATATTT                   242
AbMS14413      AATGAGCAAATCCCCTTAAACTGAAGCGCATAAGGGGATTTATATTT                   242
AbABF9692      AATGAGCAAATCCCCTTAAACTGAAGCGCATAAGGGGATTTATATTT                   242
AbDT-Ab003     AATGAGCAAATCCCCTTAAACTGAAGCGCATAAGGGGATTTATATTT                   242
```

Supplementary Table ST1
Read count table:

Sample	Raw		Cleaned			
	Reads	Bases	Unpaired	Paired	Bases	% Bases
AbDS002 (Rep1)	18,768,553	1,407,641,475	131,901	17,783,887	1,330,517,603	94.5%
AbDS002 (Rep2)	12,859,883	964,491,225	71,618	10,923,965	817,326,276	84.7%
AbDS002 (Rep3)	15,024,771	1,126,857,825	98,425	13,380,808	1,000,751,448	88.8%
AbHS002 (Rep1)	18,284,242	1,371,318,150	113,255	15,433,741	1,154,486,937	84.2%
AbHS002 (Rep2)	14,569,394	1,092,704,550	104,342	12,773,498	955,314,638	87.4%
AbHS002 (Rep3)	24,033,885	1,802,541,375	351,484	23,564,253	1,757,435,756	97.5%

Supplementary Table ST2

List of down-regulated genes

Gene	log2FoldChange	padj	Description
gene3096	-1.901	0.005	acyl-CoA dehydrogenase
gene221	-1.845	0.158	VOC family protein
gene516	-1.836	0.012	prepilin-type cleavage/methylation domain-containing protein
gene2131	-1.747	0.042	hypothetical protein
gene2892	-1.734	0.238	iron-containing redox enzyme family protein
gene2044	-1.712	0.309	four-helix bundle copper-binding protein
gene1629	-1.686	0.362	trehalose-phosphatase
gene1628	-1.607	0.482	trehalose-6-phosphate synthase
gene2783	-1.569	0.002	TetR family transcriptional regulator
gene2895	-1.541	0.368	hypothetical protein
gene2893	-1.521	0.310	damage-inducible protein CinA
gene2293	-1.463	0.362	hypothetical protein
gene2888	-1.439	0.309	hypothetical protein
gene573	-1.403	0.020	DUF805 domain-containing protein
gene1171	-1.397	0.007	hypothetical protein
gene3187	-1.384	0.000	acyl-CoA desaturase
gene2088	-1.378	0.000	O-methyltransferase
gene2278	-1.374	0.004	Metallopeptidase
gene2891	-1.370	0.426	catalase HPII
gene2894	-1.352	0.435	hypothetical protein
gene2493	-1.345	0.354	hypothetical protein
gene198	-1.336	0.056	hypothetical protein
gene222	-1.324	0.003	quaternary ammonium transporter
gene1253	-1.292	0.002	hypothetical protein
gene462	-1.290	NA	hypothetical protein
gene2377	-1.286	0.128	hypothetical protein
gene1974	-1.285	0.100	hypothetical protein
gene2556	-1.284	NA	ferredoxin family protein

gene2327	-1.282	0.080	D-amino acid dehydrogenase
gene996	-1.265	0.000	D-amino acid dehydrogenase small subunit
gene2590	-1.262	0.109	hypothetical protein
gene2499	-1.261	0.142	hypothetical protein
gene2008	-1.254	0.377	OmpA family protein
gene877	-1.243	0.095	GlsB/YeaQ/YmgE family stress response membrane protein
gene491	-1.240	0.181	Hemerythrin
gene2866	-1.217	0.045	glutathione transferase
gene1381	-1.215	0.031	Phosphatidylglycerophosphatase
gene2745	-1.149	0.015	polyisoprenoid-binding protein
gene2047	-1.148	NA	hypothetical protein
gene2058	-1.128	0.547	LysE family translocator
gene240	-1.128	0.037	Peroxiredoxin
gene2260	-1.120	0.042	universal stress protein
gene1726	-1.110	0.007	VOC family protein
gene2067	-1.106	0.430	hypothetical protein
gene1592	-1.101	0.169	hypothetical protein
gene2246	-1.097	0.507	hypothetical protein
gene2567	-1.093	0.200	MBL fold metallo-hydrolase
gene2844	-1.086	0.624	taurine ABC transporter ATP-binding subunit
gene702	-1.067	0.041	hypothetical protein
gene2014	-1.062	0.164	hypothetical protein
gene2867	-1.060	0.077	glutathione-dependent disulfide-bond oxidoreductase
gene934	-1.058	0.012	Oxidoreductase
gene2890	-1.058	0.211	NAD(P)-dependent oxidoreductase
gene2356	-1.044	0.120	hypothetical protein
gene611	-1.032	0.093	Hydrolase
gene653	-1.029	0.108	hypothetical protein
gene2852	-1.028	0.109	cytochrome ubiquinol oxidase subunit I
gene998	-1.027	0.000	RidA family protein
gene3014	-1.023	0.037	hypothetical protein
gene2087	-1.016	0.042	universal stress protein

gene2134	-1.016	0.083	hypothetical protein
gene1176	-1.011	0.049	serine hydrolase family protein
gene3188	-1.010	0.000	ferredoxin reductase
gene519	-1.007	0.014	LemA family protein
gene997	-1.005	0.007	alanine racemase
gene2911	-1.000	0.531	indolepyruvate ferredoxin oxidoreductase family protein

Supplementary Table ST3:

List of up-regulated genes:

Gene	log2FoldChange	padj	Description
gene862	1.001	0.069	membrane protein insertion efficiency factor YidD
gene395	1.009	0.101	50S ribosomal protein L18
gene391	1.014	0.080	preprotein translocase subunit SecY
gene1561	1.016	0.111	flavin reductase
gene2944	1.029	NA	4-carboxymuconolactone decarboxylase
gene595	1.030	0.128	ATP-binding protein
gene29	1.031	0.007	co-chaperone GroES
gene974	1.051	0.013	GntR family transcriptional regulator
gene86	1.053	0.009	hypothetical protein
gene1142	1.059	0.006	general secretion pathway protein
gene2768	1.066	NA	fimbrial protein
gene2207	1.077	0.000	RNA chaperone Hfq
gene3089	1.084	0.037	30S ribosomal protein S2
gene399	1.088	0.050	50S ribosomal protein L5
gene3088	1.092	0.037	elongation factor Ts
gene386	1.100	0.071	DNA-directed RNA polymerase subunit alpha
gene388	1.103	0.114	30S ribosomal protein S11
gene1691	1.127	0.037	elongation factor G
gene401	1.127	0.044	50S ribosomal protein L14
gene400	1.131	0.038	50S ribosomal protein L24
gene597	1.141	0.045	TetR/AcrR family transcriptional regulator
gene398	1.183	0.045	30S ribosomal protein S14
gene397	1.185	0.037	30S ribosomal protein S8
gene3039	1.198	0.009	C4-dicarboxylate transporter
gene402	1.205	0.070	30S ribosomal protein S17
gene1555	1.213	NA	hypothetical protein
gene1267	1.216	0.001	aminoacetone oxidase family FAD-binding enzyme
gene412	1.256	0.062	30S ribosomal protein S10
gene387	1.258	0.045	30S ribosomal protein S4

gene168	1.270	0.000	alanine:cation symporter family protein
gene403	1.292	0.044	50S ribosomal protein L29
gene977	1.332	0.020	Fe/S-dependent 2-methylisocitrate dehydratase AcnD
gene169	1.444	0.000	hypothetical protein
gene1762	1.444	0.000	hypothetical protein
gene1361	1.452	0.000	TonB-dependent siderophore receptor
gene411	1.455	0.028	50S ribosomal protein L3
gene408	1.469	0.044	50S ribosomal protein L2
gene407	1.490	0.035	30S ribosomal protein S19
gene2140	1.497	0.012	50S ribosomal protein L9
gene3040	1.500	0.044	hypothetical protein
gene404	1.512	0.020	50S ribosomal protein L16
gene409	1.515	0.008	50S ribosomal protein L23
gene406	1.543	0.015	50S ribosomal protein L22
gene410	1.547	0.009	50S ribosomal protein L4
gene292	1.580	0.006	tRNA guanosine(34) transglycosylase Tgt
gene2141	1.584	0.009	30S ribosomal protein S18
gene1162	1.610	0.000	DNA transfer protein p32
gene901	1.634	0.000	hypothetical protein
gene405	1.663	0.014	30S ribosomal protein S3
gene2142	1.664	0.001	30S ribosomal protein S6
gene1163	1.966	0.003	DNA transfer protein p32
gene970	2.102	0.184	transcriptional regulator LldR
gene972	2.249	0.106	D-lactate dehydrogenase
gene1560	2.582	0.000	methionine synthase
gene971	2.608	0.109	alpha-hydroxy-acid oxidizing enzyme
gene969	2.934	0.109	L-lactate permease
gene1559	3.249	0.006	DUF1852 domain-containing protein
